# Proteome Serological Determination of Tumor-Associated Antigens in Melanoma

**DOI:** 10.1371/journal.pone.0005199

**Published:** 2009-04-17

**Authors:** Michael Forgber, Uwe Trefzer, Wolfram Sterry, Peter Walden

**Affiliations:** Department of Dermatology, Venerology and Allergy, Charité -Universitätsmedizin Berlin, Humboldt University, Berlin, Germany; Tufts University, United States of America

## Abstract

Proteome serology may complement expression library-based approaches as strategy utilizing the patients' immune responses for the identification pathogenesis factors and potential targets for therapy and markers for diagnosis. Melanoma is a relatively immunogenic tumor and antigens recognized by melanoma-specific T cells have been extensively studied. The specificities of antibody responses to this malignancy have been analyzed to some extent by molecular genetic but not proteomics approaches. We screened sera of 94 melanoma patients for anti-melanoma reactivity and detected seropositivity in two-thirds of the patients with 2–6 antigens per case detected by 1D and an average of 2.3 per case by 2D Western blot analysis. For identification, antigen spots in Western blots were aligned with proteins in 2-DE and analyzed by mass spectrometry. 18 antigens were identified, 17 of which for the first time for melanoma. One of these antigens, galectin-3, has been related to various oncogenic processes including metastasis formation and invasiveness. Similarly, enolase has been found deregulated in different cancers. With at least 2 of 18 identified proteins implicated in oncogenic processes, the work confirms the potential of proteome-based antigen discovery to identify pathologically relevant proteins.

## Introduction

The antigenicity of tumor cells as defined by antibodies in the sera of cancer patients may provide new insights into the interrelationship of tumor and immune system, and into the molecular pathology of the tumor cells. It, thus, may guide the development of new immune intervention strategies for therapy and lead to new markers for diagnosis and disease monitoring [1]–[8]. The most often employed approach to the identification of serologically defined antigens so far has been the serological identification of recombinant expression cloning (SEREX) approach [9]–[12]. Hereby, cDNA libraries of tumor tissue, tumor cell lines or human testis are cloned into λ phage expression systems and screened with sera of cancer patients. SEREX has been employed to identify serological antigens for melanoma and the SEREX database (www2.licr.org/CancerImmunomeDB) lists 102 melanoma-associated antigens [9], [12], [13]. These antigens can be categorized as differentiation antigens such as tyrosinase, cancer-testis antigens such as MAGE-1 or NY-ESO, overexpressed gene products, mutated gene products such as cdk4 or p53 mutants, and cancer-related autoantigens such as CEBP-γ.

In addition to mutation, over- or ectopic expression, antigenicity of a protein expressed by tumor cells may be determined by post-translational modifications or altered accessibility. Proteome-based approaches may complement SEREX approaches in these aspects and, by displaying and screening with patient sera the proteomes of tumors in single 2-dimensional electrophoresis gels, provide information on the scope of tumor cell antigenicity. This technology has been applied to renal cell carcinoma, ovarian cancer, pancreatic adenocarcinoma, prostate cancer, gastric cancer, hepatocellular carcinoma, colon cancer and lung squamous carcinoma [4], [14]–[29]. Using technologies developed for proteomics research, the antigens are identified by matching antigen spots in Western blots with protein spots in replica silver-stained gels, excising these protein spots and identifying the antigens by peptide mass fingerprint (PMF), peptide fragment fingerprint (PFF) or *de novo* peptide sequencing of tryptic fragments of the proteins [30]. This approach has variously been named proteome serology [31], immunoproteomics [16], serological proteome analysis (SERPA) and other [21]. We employed this combination of Western blot analysis of serological specificities and mass spectrometry-based protein identification to determine the range of the immunogenicity of melanoma and the specificities of the corresponding antibody responses in the patients.

## Results

### Seroreactivities for melanoma-associated antigens

**Figure 1:**
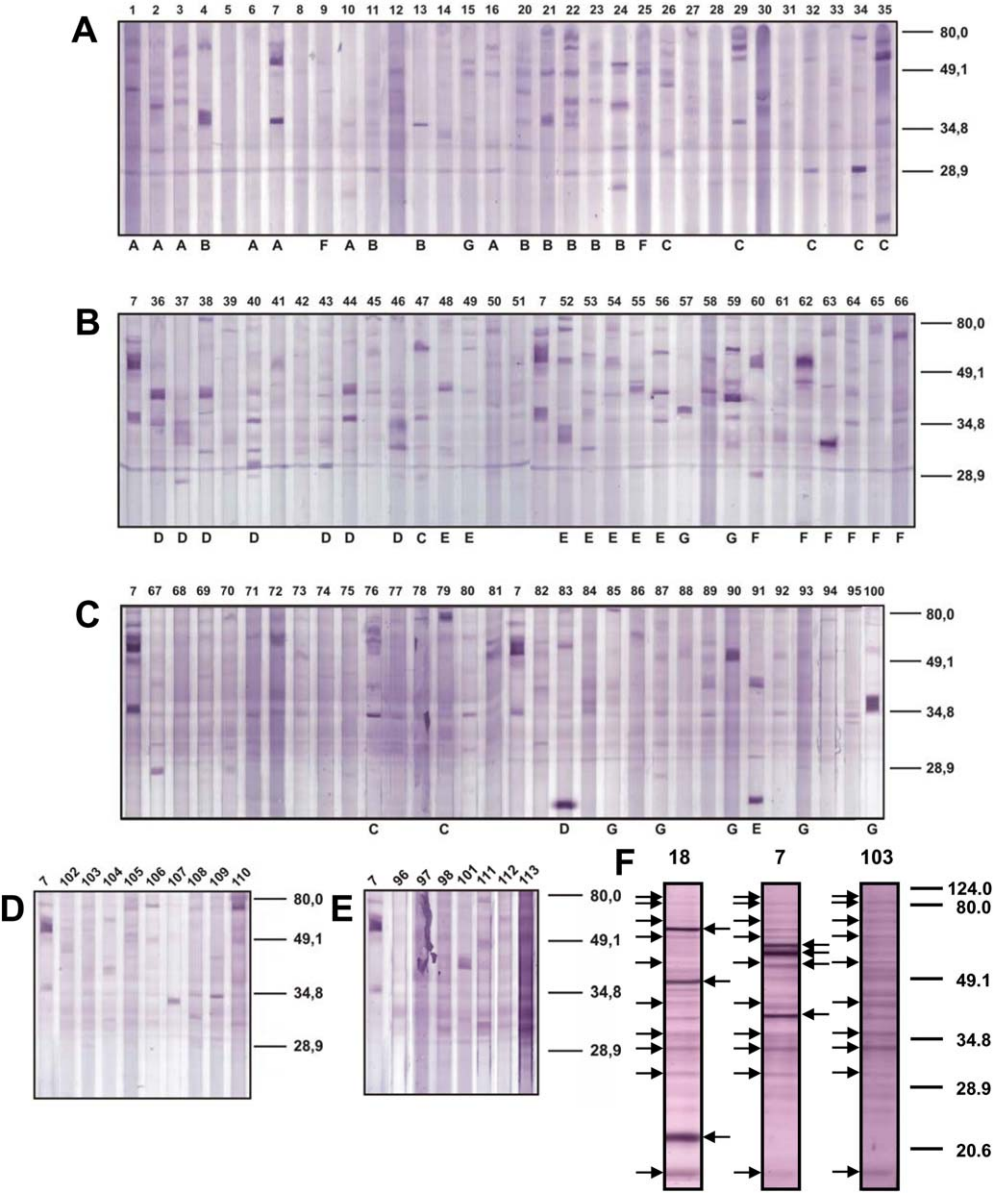
Pattern of seroreactivities of melanoma patients, healthy controls and patients with other diseases against the melanoma cell line M-NRT. Total protein extract of the tumor cells were separated by SDS-PAGE, blotted onto nitrocellulose and probed with the sera of the melanoma patients (Panels A–C), of healthy control donors (Panel D, sera 102–110) or patients with cutaneous lymphoma (Panel E, sera 96–98), pancreas carcinoma (Panel E, serum 101) or visceral leishmaniasis (Panel E, sera 111–113). The numbers atop of each lane represent the number of the sera and are used throughout this report. For comparison, melanoma serum 7 was included in all blots. The Western blot analyses were done with the sera at a dilution of 1/6 in a multiple channel blotting/Western blot developing chamber for panels A–E. The letters underneath the lanes indicated the combinations of sera for the multiple probing of the 2D Western blots shown in Figure 2 and summarized in Table 1. Panel F: High-resolution 1D Western blot for comparison of the seroreactivities of melanoma patients and a healthy donor against the melanoma cell line M-NRT. In contrast to the blots shown in panels A–E, the sera were applied 1/200 to isolated lanes from SDS-PAGE in sealed plastic bags which results in a better definition of the bands when compared to blots from multiple blotting devices. Serum 18 is autologous to tumor cell line M-NRT, serum 7 is from a different patient and serum 103 from a healthy donor. The arrows to the left of the lanes indicated antigen bands that are shared between the patients and the healthy controls and occur in every blot. The arrows to the right of the lanes indicated prominent antigen that are detected only by the sera of melanoma patients.

To determine the frequencies and specificity patterns of antibody responses to melanoma-associated antigens, we tested by Western blot analyses the reactivities of sera of 94 patients with melanoma (see Table S1 for clinical details of the cases) against protein extracts from the melanoma cell line M-NRT separated by 1-dimensional SDS-PAGE (Figure 1A–C). As controls, the sera of 9 healthy individuals (Figure 1D), and of patients with cutaneous lymphoma, pancreas carcinoma or visceral leishmaniasis, an infectious disease that is known to induce responses to autoantigens of the infected host, were tested (Figure 1E). Overall, the signals were relatively weak given the high serum concentrations of 1∶6 used for these Western blots. A large number of faint background bands were detected with the sera of the healthy controls and patients alike as illustrated with Figure 1F which shows the Western blots with serum of a healthy donor (Serum 103) and the sera of two melanoma patients (Serum numbers 7 and 18). The Western blots shown in Figure 1F were developed as individual strips in separate plastic bags at a dilution of 1 in 200 which results in a better definition of the bands but is not suited for comparative screening of large numbers of sera. The arrows to the left of the Western blot lanes indicate shared signals found with patient and control sera alike. The arrows to the right of the lanes are stronger and indicate unique bands seen only with sera of melanoma patients. Serum 18 was from the same patient from whose tumor the melanoma cell line M-NRT had been established. This Western blot, thus, documents the reactivity in the autologous combination of tumor cells and serum. All other sera were from different patients or healthy donors, thus displaying the reactivity in heterogeneous combination of tumor cells and serum. The antigens thus detected are, therefore, expected to be antigens shared between the tumors of the serum donors and the test tumor cells M-NRT. With nearly two-thirds of the sera of the melanoma patients prominent bands are detectable that are not found with the healthy control sera or that are much stronger than those in the controls (Figure 1). The numbers of such prominent antigens detected with the reactive sera ranged between 2 and 6 per patient serum. Their masses were between 21 and 90 kDa with the bulk of the stronger bands between 40 and 80 kDa. The patients whose sera were tested represent, with the exception of ocular melanoma, all forms of melanoma. The majority of the patients were at stage 3 or 4 of disease but some were at earlier stages (Table S1). The course of disease ranged from relatively slow progression to aggressive disease. Within this range of patients there is no correlation of the numbers of antigens targeted, and frequencies or pattern of seropositivity with the clinical conditions. A few stronger reactivities were seen with the sera of healthy controls and patients with lymphoma, pancreas carcinoma or Leishmaniasis. These antigens were different than those detected with the sera of melanoma patients. In summary, about two-thirds of the patients had developed prominently detectable antibody responses against melanoma-associated antigens. The serospecificity patterns, however, were heterogeneous with no antigen that induced responses in a majority of the patients.

### Identification of melanoma-associated antigens that raise antibody responses

**Figure 2:**
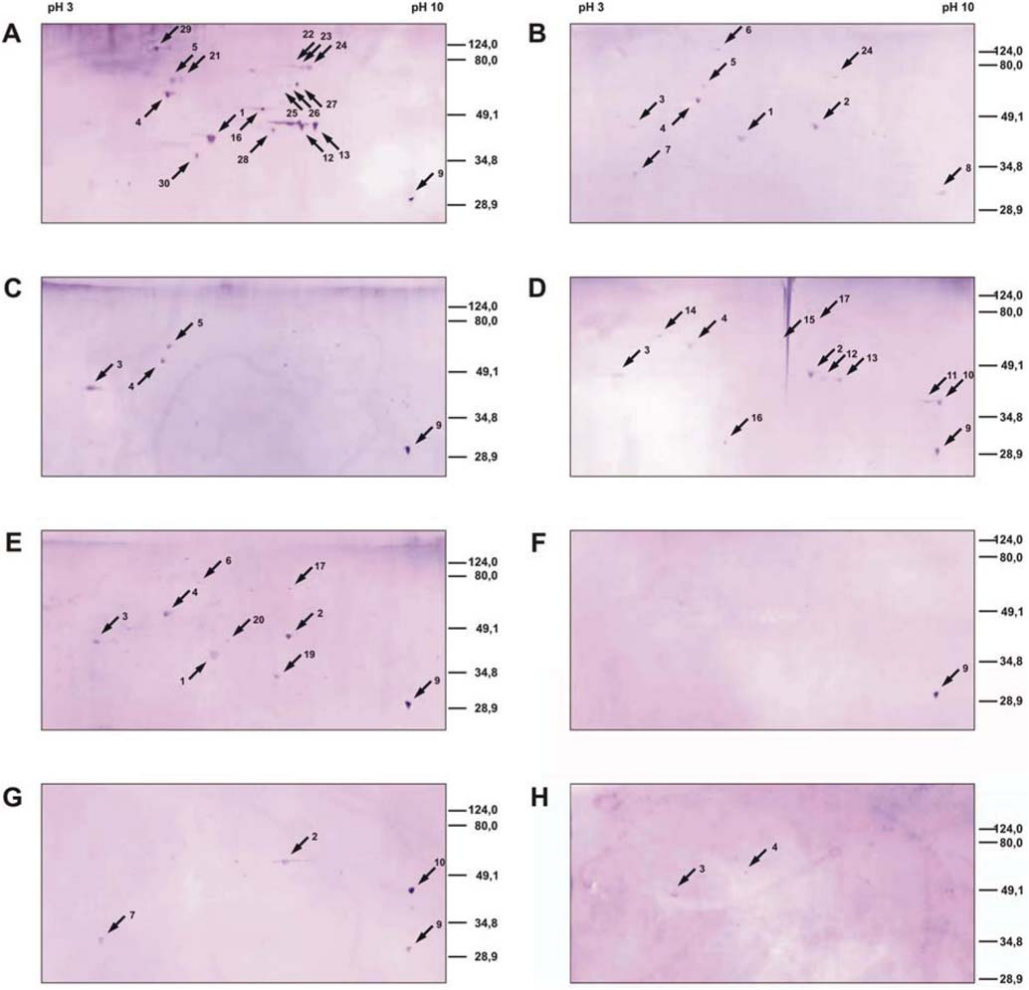
Seroreactivities of melanoma patients and healthy controls against antigens of the melanoma cell line M-NRT isolated by 2DE. Total protein extracts of M-NRT cells were separated with an isoelectric focusing pH range of 3–10 in the first and SDS-PAGE in the second dimension, blotted onto nitrocellulose membranes and probed with the sera of the patients as listed in Table 1. Serum dilutions were 1/200. The blots shown in Panels A through G were probed successively with 8 different patient sera each, blot H was probed with the sera of 9 healthy controls. The arrows indicated the antigens that could be assigned to protein spots in the silver-stained gel show with Figure 3. The numbering for the antigens is used throughout this report.

Direct identification of antigens from 1-dimensional SDS-PAGE gels is not possible because of the complexity of the protein mixtures at every position in the lanes and difficulties in exactly aligning Western blots with silver-stained gels. We, therefore, separated the proteins of the melanoma cell line M-NRT by 2-dimensional electrophoresis with a pH gradient of 3 to 10 in the isoelectric focusing gels. Nine replica gels were prepared, eight used for Western blot analyses (Figure 2, panels A–H) and one for silver staining (Figure 2). Each of the Western blot filter A through G were probed successively with the sera of 8 melanoma patients, 56 sera in all. These are all available sera with which prominent bands were detected by 1DE. Blot H was tested with the sera of 9 healthy controls. The use of multiple sera per Western blot is required to expose several antigen spots per blot which is essential for spot pattern recognition and alignment of silver-stained proteins with Western blot spots. The serum combinations for multiple probing were selected to allow good pattern definition for assigning Western blot spots with protein spots in the silver-stained gel, and are listed in Table 1 where the numbering of the sera corresponds to the numbering in Figure 1. The letters underneath the blot lanes in Figure 1 refer to the 2D Western blots in Figure 2.

**Table 1:** Serum combinations for 2-dimensional Western blot analyses and numbers of identified antigens.

Western blot # †	Sera‡	Number of antigens#			Spot numbers of the identified antigens*
		In the Western blots	Assigned to proteins	Identified	
A	1, 2, 3, 6, 7, 10, 16, 18	26	17	14	1,4,5,9,12,13,21,23,24,27,28,29,30,31
B	4, 11, 13, 20, 21, 22, 23, 24	19	9	7	1,2,3,4,5,8,24
C	26, 29, 32, 34, 35, 47, 76, 79	21	4	4	3,4,5,9
D	36, 37, 38, 40, 43, 44, 46, 83	20	12	9	2,3,4,9,10,11,12,13,16
E	48, 49, 52, 53, 54, 55, 56, 91	24	9	6	1,2,3,4,9,19
F	9, 25, 60, 62, 63, 64, 65, 66	3	1	1	9
G	15, 57, 59, 85, 87, 90, 93, 100	15	4	3	2,9,10
H	102–110	4	2	2	3,4

† The numbering refers to the Western blots shown in Figure 2.

‡ The serum numbering refers to the serum numbers in Figure 1.

# The numbers of antigens correspond to the number of spots detected in the Western blots of Figure 2, of those assigned to protein spots in the silver-stained gel shown with Figure 3 and the number of the assigned antigens that could be identified.

**Figure 3:**
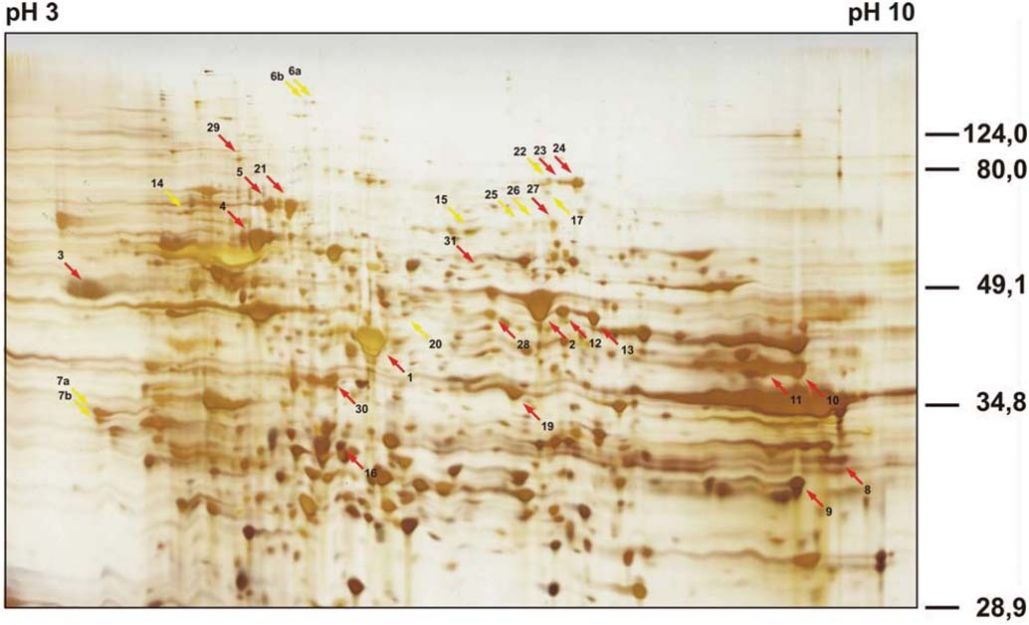
Identification of melanoma-associated antigens detected by sera of melanoma patients. The protein spots that could be assigned to antigen spots in the Western blots (arrows here and in Figure 2) were excised from the gels and treated with trypsin. The resulting fragments were analyzed by mass spectrometry to identify the antigens. Eighteen different antigens were identified that were found in 46 different spots in the Western blots shown in Figure 2.

* The spot numbers correspond to the numbers of assigned antigen and protein spots indicated with arrows and numbered in Figures 2 and 3.

The antigen signals were weak but clearly detectable in the 2-dimensional Western blots (Figure 2). The blots display 26, 19, 21, 20, 24, 3 and 15 antigen spots in panels A through G, respectively, altogether 128 antigen spots. With the sera of the healthy controls, 4 antigen spots were detected (Figure 2H). The antigens concur in numbers and mass distribution with the antigenicity patterns shown in Figure 1. Fifty-eight of the antigens could be assigned to protein spots in the silver-stained gels, 17, 9, 4, 12, 9, 1, 4 and 2 for the blots in Figure 2A through H, respectively. The antigen-protein assignments were done by first aligning gel and Western blots by their geometry as defined with artificial marker proteins spotted on cardinal points of the gels and marker protein spots detected in the blots after Ponceau S staining. A number of prominent marker spots were mapped for confirmation by partial blotting and matching the corresponding Western blots and silver-stained gels. Then, the spot patterns in the local environments of the antigens were compared taking spot sizes and shapes into consideration. The assigned and identified spots are indicated with arrows and numbered in the Western blots and the corresponding silver-stained gel shown in Figures 2 and Figure 3. The protein spots corresponding to the assigned antigens were excised from the gel, destained and subjected to trypsin digest. The resulting tryptic fragments were analyzed by peptide mass fingerprint by MALDI-TOF-MS with MASCOT and PROWL analyses of the peak lists. Forty-six of the 58 assigned antigens could, thereby, be identified. Spots number 10, 12, 13 and 24 were found in 2 Western blots, spots 1 and 5 in 3, spot 2 in 4, spot 3 in 5 and spots 4 and 9 in 6. All other assigned antigens were found only once. The 46 antigen spots, thus, represent 18 different antigens.

**Figure 4:**
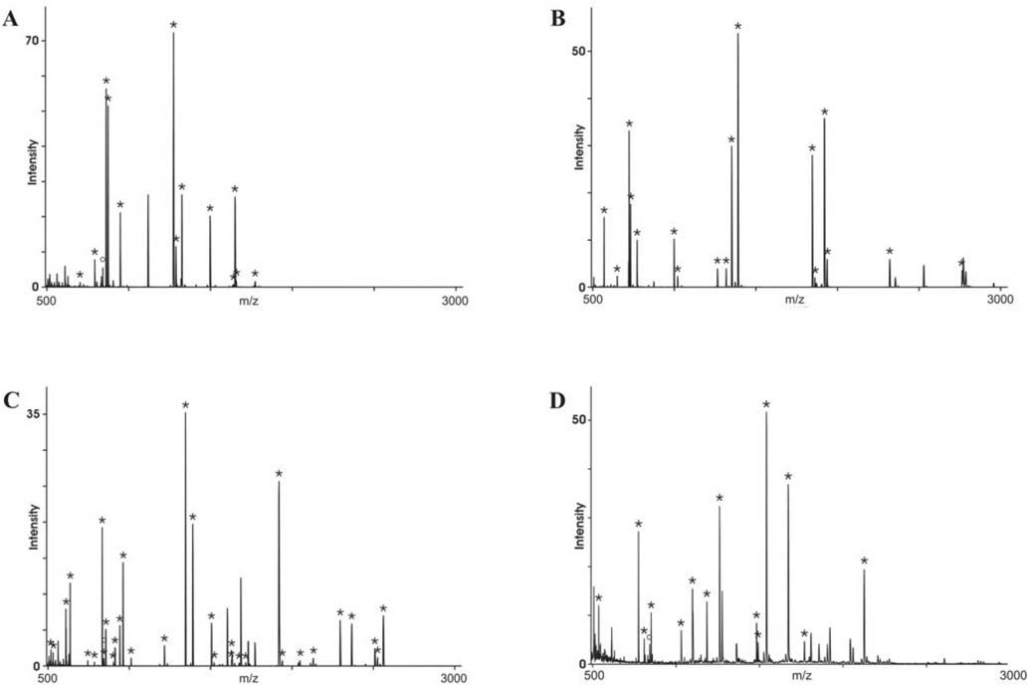
Peptide mass fingerprint spectra of antigens identified by mass spectrometry. Panel A shows the mass spectrum of the tryptic fragments for antigen spot number 9 identified as galectin-3, panel B the respective spectrum for antigen spot 1 identified as the actin filament capping protein MCP, panel C antigen spot 4 identified as the heat shock protein HSP60 and panel D antigen spot 28 identified as the elongation factor EF-Tu. The peptide mass fingerprint spectra for the other 17 antigen spots are provided with the supplementary materials indicated in Table 2 together with the statistics of the mass-spectrometric identification of the proteins. Asterisks indicate the tryptic fragment masses matched to the database sequences of the proteins.

As examples for the mass-spectrometric identification of the antigens, the fingerprint mass spectra for spot number 9 identified as galectin-3, spot number 1 identified as the gelsolin-like actin filament-capping protein MCP, spot number 4 identified as heat shock protein 60 (HSP 60) and spot number 28 identified as the elongation factor EF-Tu are shown in Figure 4A, B, C and D, respectively. The spectra for the other antigens are provided as supplementary materials (Figures S1, S2, S3, S4, S5, S6, S7, S8, S9, S10, S11, S12, S13, S14, S15, S16, S17) as indicated in Table 2 together with the gene identifier numbers, protein-chemical parameter and the results of the mass-spectrometric identification for all antigens. In the cases where masses remained that could not be assigned to the identified proteins, a secondary peptide mass fingerprint analysis was done with these remaining masses. In no case could a second protein be identified indicating that the analyzed spots did not contain major contaminating proteins. The identified antigens can be grouped according to their cell biological functions into different categories. Three of the antigens are heat shock proteins (HSP60, HSP70 and HSP70 protein 9B), 7 are enzymes of the cellular metabolism (enolase I, dienoyl-CoA reductase, aldolase A, fumarate hydratase, aldose reductase, aconitase and lactate dehydrogenase), hnRNP-1 is a nuclear protein involved in RNA processing, EF-Tu an elongation factor in protein biosynthesis, MCP affiliated with the cytoskeleton and involved in cell migration, calumenin a calcium-binding protein involved in regulation of metabolic processes, VCP participates in the regulation of protein export and the organization of the Golgi apparatus, LAP3 and PSME1 are involved in protein metabolism, and galectin-3 is a lectin with specificity for galactose. The sequence coverage of the mass-spectrometric fingerprint analysis of HnRNP is somewhat low. However, HnRNP had been repeatedly identified from different gels with better sequence coverage so that we are sure of its correct identification. For all other antigens the sequence coverage is sufficiently high for unequivocal identification of the corresponding proteins. Galectin is extracellularly expressed and involved in various interactions with serum proteins, other cells and extracellular matrix, and variously implicated in cancer-related processes such as metastasis formation and invasiveness. Enolase I, although a cytosolic protein, has been reported to be exported and is found in the extracellular environment of some tumors. Of the identified antigens, galectin-3 and HSP60 most often induce antibody responses in melanoma patients followed by calumenin and enolase I. For HSP60 and calumenin seroreactivity was also detected in healthy donors tested.

**Table 2:** Antigens recognized by sera of melanoma patients and identified by proteome serology.

Spot #	Antigen (accession number)	M_W_ [kDa]	pI	Number of tryptic fragments		Sequence coverage	PMF spectrum Figure
				total	assigned		
1	gelsolin-like actin filament-capping protein MCP	38.5	5.88	26	17	42%	4
	gi|63252913						
2	enolase 1	47.1	7.01	76	28	59%	S1
	gi|29792061						
3	calumenin	37.1	4.47	76	22	53%	S2
	gi|2809324						
4	heat shock protein HSP60	61.0	5.70	52	32	57%	4
	gi|31542947						
5	heat shock protein HSP70 protein 8	70.9	5.37	84	36	49%	S3
	gi|5729877						
8	2,4-dienoyl-CoA reductase	35.8	9.50	40	12	31%	S4
	gi|1575000						
9	galectin-3	26.2	8.58	55	13	44%	4
	gi|12654571						
10	aldolase A	39.4	8.49	87	26	62%	S5
	gi|49456715						
11	aldolase A	39.4	8.49	56	21	53%	S6
	gi|4930291						
12	fumarate hydratase	54.6	8.85	82	26	41%	S7
	gi|32880021						
13	fumarate hydratase	54.6	8.85	76	33	47%	S8
	gi|32880021						
16	proteasome activator subunit 1 PSME1	28.7	5.78	55	20	57%	S9
	gi|5453990						
19	aldose reductase	35.7	6.56	65	17	33%	S10
	gi|493797						
21	heat shock protein HSP70 protein 9B	73.6	5.87	61	19	33%	S11
	gi|24234688						
23	aconitase 2	84.0	7.20	61	38	47%	S12
	gi| 5304852						
24	aconitase 2	85.5	7.62	82	50	57%	S13
	gi|20072188						
27	nuclear protein HnRNP1	60.1	6.65	49	12	23%	S14
	gi| 52632383						
28	elongation factor EF-Tu	49.5	7.70	52	17	35%	4
	gi|55584035						
29	valosin-containing protein VCP	89.3	5.14	43	24	28%	S15
	gi|6005942						
30	LDH H	36.5	5.72	96	12	37%	S16
	gi|13786847						
31	leucine aminopeptidase LAP3	56.0	7.58	84	28	56%	S17
	gi|4335941						

## Discussion

About two thirds of the 94 melanoma patients tested in this study had mounted specific antibody responses against melanoma-associated antigens with an average of about 3 antigens detectable per seropositive patient. Only a few of the specific antigen bands were shared between different patients and no two patients displayed the same pattern of antigenicity. In highly resolving 2-dimensional Western blots 128 antigens were detected with sera of 56 patients, i.e. an average of 2 to 3 antigens per patient. Four antigens were also found with the sera of the healthy controls which corresponds to an average of less than 0.5 antigens per serum and is comparable to other studies done for cancer and infectious diseases. Fifty-six of these antigens could be assigned to protein spots in silver-stained gels and 44 of them identified by mass spectrometry. They were found to represent 18 different antigens, two, galectin-3 and HSP60, were found in 6 of the 7 Western blots, several more in between 2 and 5 blots but the majority was detected only once. The serological immunoreactivities against the tumor cells, thus, are mostly heterogeneous. Nonetheless, the majority of the patients had mounted antibody responses against several antigens of the melanoma cell line tested and some antigens raised responses in a relatively high proportion of cases. For all antigens, the signals in the Western blots were weak, despite high serum concentrations, indicating that the B cell responses were weak. However, all these responses were secondary, IgG responses, thus, depending on repeated stimulation by the antigens and induction of MHC class II-restricted CD4^+^ helper T cells with specificity for the same antigens in order to induce immunoglobulin class switch. These antigens, therefore, are expected to harbor CD4 T cell epitopes as well as B cell epitopes.

The identified antigens represent different cell-biological categories of proteins including structural proteins (MCP), metabolic enzymes (enolase 1, dienoyl-CoA reductase, aldolase A, fumarate hydratase, aldose reductase, aconitase, LDH-H), heat shock proteins (HSP60, HSP70, HSP70 protein 9B), proteins involved in protein biosynthesis and protein metabolism (HnRNP1, EF-Tu, PSMEI1, LAP3), a calcium-binding regulator protein (calumenin) and a lectin of the outer membrane (galectin-3). The prominence of metabolic enzymes among antigens targeted by serological immune responses has also been reported for patients with pancreatic, lung, renal, colon and hepatic cancers [15], [20], [23]–[25], [29]. As for the majority of other serologically defined tumor-associated antigens identified so far, there is no recognizable structural cause for the immunogenicity of the antigens (www2.licr.org/CancerImmunomeDB). As judged from the protein gels, they do not grossly deviate in molecular masses and isoelectric points from what is known for these proteins. Comparative proteomics studies have shown that some of the antigens may be overexpressed in tumor cells compared to their normal counterparts [32], [33]. Such overexpression may promote immunogenicity of these proteins in cancer patients. With two exceptions, the antigens are intracellular proteins and not directly accessible to the immune system. The one exception is galectin-3. It is very interesting that seroreactivities for this protein were found in 6 of the 7 Western blots suggesting that it is a relatively dominant antigen and, maybe suited for targeted therapy. The second, enolase, has been reported to be, under not yet understood circumstances, exported from tumor cells. For the other antigens it is likely that their immunogenicity is related to destruction of tumor cells and their aberrant exposure to the immune system.

Only one of the antigens reported herein, galectin-3, had already been reported for melanoma [12]. Other antigens such as EF-Tu, HSP70, HSP60, aldolase, fumarate hydratase, aldose reductase, aconitase, HnRNP1, EF-TU, VCP and enolase have been reported for other cancers including cutaneous T cell lymphoma, and renal, hepatic, lung and pancreatic cancers but not for melanoma [15], [20], [23]–[25], [29], [34], [35]. The remaining antigens appear to be new tumor-associated antigens. So far all serologically defined antigens known for melanoma have been identified by SEREX [9], [12], and the SEREX database lists 102 melanoma-associated antigens (www2.licr.org/CancerImmunomeDB). Besides a number of unknown function and superfamily affiliations, and some cancer/testis antigen [1], [3], [5], [10], the vast majority of the SEREX-defined melanoma-associated antigens are of the same functional categories as those reported herein: chaperones, metabolic enzymes, proteins involved in protein biosynthesis and catabolism, structural proteins and regulators of the cellular metabolism. Galectin-3, found by SEREX and in the present study by proteome serology, is of great interest as it is one of the very few serologically defined tumor-associated antigens that are present at the outer cell surface. It has been described as deregulated in different cancers and as involved in cancer-related processes such as cell plasticity and vasculogenesis [36]–[43]. Also enolase is exported and has been implicated in cancer [44]. It had been identified as tumor-associated antigen in renal cell carcinoma, lung squamous carcinoma, leukemia and pancreatic ductal adenocarcinoma [23], [24], [29], [45] (Novelli et al. WO/2008/037792). These two proteins may be targets for therapeutic intervention.

Proteome serology has been employed for antigen discovery for renal cell carcinoma [24], breast cancer [21], colon carcinoma [15], [21], prostate cancer [26], pancreatic adenocarcinoma [20], ovarian cancer [19], hepatocellular carcinoma [25], leukemia [45] and lung squamous carcinoma [23], [29]. While many of the specific antigens reported for these cancers differ from those found for melanoma, they do represent the same classes of proteins as discussed above. On the other hand, there are antigens such as galectin-3 that were found by proteome serology so far only for melanoma, and others only for other cancers. Some of these restricted antigens are ubiquitously expressed so that the basis for their restricted antigenicity remains unclear. Very interesting is the limited overlap of antigens discovered by SEREX and proteome serology. This can not be explained by differences in the sensitivity of these technologies as the more sensitive technology should include also the antigens discovered by the less sensitive approach. A possible explanation might be that these two technologies focus on different sets of antigens: SEREX readily identifies mutated antigens and antigens arising from splice variations but not post-translational modifications. Proteome serology, on the other hand, can detect proteins whose antigenicity is related to post-translational modifications but mutations only when *de novo* sequencing approaches are used instead of conventional protein identification by peptide mass fingerprint [46], [47]. The two technologies thus are complementary.

## Materials and Methods

### Tumor cells, cell lines and sera

The tumor cells were isolated from melanoma metastases by treating minced tissue with collagenase free of contaminating protease activities and DNAse, passaging the resulting suspension through a cell sieve to remove connective tissue, allowing the tumor cells to adhere to tissue culture plastic plates and wash off non-adherent cells. The tumor cells were harvested by scraping them off the surface for direct use. Melanoma cell lines were established from such primary cells by culturing in DMEM (Gibco, Heidelberg) with 10% FCS (Biochrome, Berlin) and pencillin/streptomycine (Gibco, Heidelberg) at 37°C with 8% CO_2_. The cells were processed for electrophoretic analysis immediately after harvest from the tumor nodules or from cell cultures. The sera were collected from 94 patients with melanoma at different stages of disease and 9 healthy donors of the same average age. The study with human subjects had been reviewed and approved by the institutional ethics committee of the Charité – Universitätsmedizin Berlin (Si. 277, September 11, 2003). The materials were obtained and used with written informed consent of the donors.

### Protein sample preparation

The cells were collected by centrifugation at 1,600×g for 10 minutes at RT, washed three-times with PBS and solubilized by a protocol adapted from Görg et al. [48] and Chan et al. [49] with lysis buffer (7 M urea, 2 M thiourea, 2.5% Triton X-100, 2% β-mercaptoethanol, 0.8% Pharmalyte pH 3.5–10 (LKB, Freiburg, Germany), 200 µM Pefablock® (Merck, Darmstadt), 1 µM pepstatin (SIGMA, Munich, Germany) and 10 µM leupeptin (SIGMA, Munich, Germany) by vortexing and sonication for 10 minutes in an ice cooled water bath. The cell extracts were incubated for one hour at room temperature (RT) with 4,000 U/ml benzonase (Merck, Darmstadt, Germany) to degrade nucleic acids and then centrifuged at 350,000×g at 15°C for 15 minutes. The supernatants were collected, incubated once again with Benzonase for 10 minutes at RT and cleared by ultracentrifugation as before.

### Two-dimensional gel electrophoresis (2DE)

Isoelectric focusing (IEF) was run in immobilized pH-gradient gel strips (IPG strips 180 mm×3 mm; Pharmacia, Freiburg) with a pH range of 3 to 10 [48], [49]. About 100 µg protein, extract of 1×10^6^ cells in 350 µl solubilization buffer were loaded into an IPG strip by in-gel re-swelling overnight at RT under silicon oil in a nitrogen- and water-saturated atmosphere to prevent oxidation of the protein and drying of the gel strips. The loaded strips were rinsed, mounted on a cooled ceramic plate and connected to the electrodes via water-wetted paper bridges. The IEF was run at 20°C under silicon oil in a nitrogen- and water-saturated atmosphere with 0.15 mA per IPG strip and 50 V for 18 h, 150 V for 1 h, 300 V for 2 h, 600 V for 1 h, 3,500 V for 6.5 h and 5,000 V for 3 h, a total of 40,000 Vh for pH 3–10 IPG strips. After the run, the IPG-strips were stored at −20°C. For the second dimension, IPG strips were thawed, rinsed with de-ionized water and equilibrated to SDS-PAGE conditions for 15 minutes in 6 M urea, 30% glycerol, 2% sodium dodecylsulfate (SDS), Tris pH 6.8 and bromophenol blue, 1% dithiothreitol (DTT) followed by 15 minutes in the same buffer but with 4% iodoacetamide instead of DTT. The equilibrated IPG-strips were rinsed with de-ionized water and placed gel-side to gel-side onto the 4.8% acrylamide, 0.13% bisacrylamide stacking gel of a horizontal SDS polyacrylamide gel with a 12.3% acrylamide, 0.34% bisacrylamide separation gel. The settings for the runs were 1,000 V, 40 W and 20 mA for 2–3 h for the pre-run to transfer the protein from the IPG strip into the SDS polyacrylamid gel, followed by 1,000 V, 40 W and 40 mA for the separation until the running front reached the anodic end of the gel.

### Protein staining

The gels were stained with the high-sensitivity silver staining approach by Blum and colleagues [50]. Briefly, SDS-PAGE gels were fixed in 40% Methanol, 10% acetic acid for one hour or overnight. Then, the gels were washed three times for 20 minutes in de-ionized water, sensitized for one minute in 0.02% sodium thiosulfate, washed three times for 20 seconds in water and incubated for 20 minutes in silver staining solution (0.2% silver nitrate, 0.0074% formaldehyde). After washing three times for 20 seconds in water, the gels were incubated in developing solution (6% sodium carbonate, 0.00015% formaldehyde, 0.0004% sodium thiosulfate) until protein spots became visible. The reactions were stopped with 0.025% EDTA in water.

### Western blots

The proteins from unstained SDS-PAGE were transferred onto nitrocellulose membranes (Schleicher & Schüll, Dassel, Germany) by semi-dry blotting for 2 hours at 400 mA. Free binding sites on the membranes were blocked with 5% low fat milk powder in Tris-buffered saline (TBS) for one hour at room temperature or at 4°C overnight. After blocking, the membranes were incubated with patient sera at a 200-fold dilution for one hour at room temperature, washed thrice for 10 minutes with TBS and incubated for 30 min with alkaline phosphatase-labeled anti human IgG (Anti-Human Ig-AP, Fab fragment; Boehringer Mannheim) at a 5,000-fold dilution. After washing three times 10 minutes with TBS, the membranes were equilibrated to developing buffer (100 mM NaCl, 100 mM Tris-HCl, pH 9.5) and developed in the dark with 100 µl BCIP and 100 µl NBT in 100 ml developing buffer until antigen spots became visible. The reactions were stopped by replacing the developing solution with water. For 1-dimensional Western blots, the proteins of the melanoma cells were separated by SDS-PAGE, 12% acrylamide, 0.8% bisacrylamide, blotted 1 h with 60 mA onto nitrocellulose and processed as above. For detection of antigens, the sera were applied in a multiple channel device (Figure 1A–E) or, after cutting the blots into strips, in individual sealed plastic bags (Figure 1F). Since efficient agitation of the serum solutions is not possible in the multiple channel device and antigen binding is solely dependent on diffusion, the sera had to be employed at a high 6-fold dilution. For the blots developed as strips in individual plastic bags, 200-fold dilutions of the sera were used as for the 2D Western blots.

### Matching antigens and protein spots

To match antigen spots in Western blots with the corresponding protein spot in the silver-stained gel, the coordinates of the blots and the gel were defined, first, with artificial spots at the corner points, Ponceau S staining of the blot filter and aligning the spot pattern with the spot pattern of the silver-stained gel and partial blotting of a master gel and definition of marker spot sets, second, definition of spot pattern in the local environments of the antigen spots to match blots and gels in these local regions accurately, third, comparing the sizes and shapes of the antigen and protein spots and considering only those that are alike in these two parameter.

### In-gel digestion of protein

The protein spots were excised manually with a self-made spot picker and de-stained as described by Gharadaghi and colleagues [51] with 50 µl of Farmer's reducing solution (15 mM potassium ferricyanide and 50 mM sodium thiosulfate, both dissolved in water), then washed three times for 5–10 minutes with 150 µl water. Then the gel spots were soaked in acetonitrile and dried under vacuum. The gel pieces were re-swollen in 7.5 µl of 5 mM ammonium bicarbonate with 75 ng of modified porcine trypsin (sequencing grade, modified; Promega; Madison, USA) to fragment the protein. After 10 minutes, 7.5 µl of 5 mM ammonium bicarbonate were added and the solution with the gel pieces incubated for at least 4 hours in a 37°C water bath. For MS analysis, 1.5 µl of the aqueous supernatants were mixed with 1 µl of 2,5-dihydroxybenzoic acid (DHB) (SIGMA, Munich, Germany) (5 mg/ml water) directly on MALDI targets (MTP AnchorChip 600/384, Bruker Daltronik, Bremen) and air-dried.

### Mass spectrometry

Mass spectrometric measurements were made with a Reflex IV MALDI-TOF mass spectrometer (MS; Bruker Daltonik, Bremen) in reflector mode at an acceleration voltage of 20 kV. The MS was calibrated either with the external standards angiotensin II (1046.5 Da), angiotensin I (1296.6 Da), bombesin (1619.8 Da), substance P (1347.7 Da), ACTH 1–17 (2093.0 Da) and ACTH 18–39 (2465.1 Da) or with the autolytic 842.50 Da and 2211.10 Da trypsin fragments as internal standards. Monoisotopic peptide masses were recorded. The spectra were processed by the “Xmass” software (Bruker Daltonik, Bremen) and the peaks annotated automatically and checked manually. Post-source decay (PSD) analyses were done in 10 sections for the entire mass range and data accumulated from up to 300 shots per section. The peak lists of the mass spectra were the basis for peptide mass fingerprint analyses with the Mascot software (Matrix Science; http:www.matrixscience.com/search_form_select.html) and profound (prowl; http://prowl.rockefeller.edu/profound_bin/WebProFound.exe) using the NCBI sequence database.

## Supporting Information

Table S1 Patient data for the sera used in the study. Melanoma patients and sera used for proteome-serological analysis of the tumor-associated antigenicity in melanoma.
(0.22 MB DOC)

Figure S1 Peptide mass fingerprint spectrum of the protein in gel spot 2: Enolase 1. Asterisks indicate fragment masses assigned to the identified protein.
(2.40 MB TIF)

Figure S2 Peptide mass fingerprint spectrum of the protein in gel spot 3: Calumenin. Asterisks indicate fragment masses assigned to the identified protein.
(2.40 MB TIF)

Figure S3 Peptide mass fingerprint spectrum of the protein in gel spot 5: HSP70 protein B. Asterisks indicate fragment masses assigned to the identified protein.
(2.32 MB TIF)

Figure S4 Peptide mass fingerprint spectrum of the protein in gel spot 8: 2,4-dienoyl-CoA reductase. Asterisks indicate fragment masses assigned to the identified protein.
(2.33 MB TIF)

Figure S5 Peptide mass fingerprint spectrum of the protein in gel spot 10: Aldolase A. Asterisks indicate fragment masses assigned to the identified protein.
(2.34 MB TIF)

Figure S6 Peptide mass fingerprint spectrum of the protein in gel spot 11: Aldolase A. Asterisks indicate fragment masses assigned to the identified protein.
(2.32 MB TIF)

Figure S7 Peptide mass fingerprint spectrum of the protein in gel spot 12: Fumarate hydratase. Asterisks indicate fragment masses assigned to the identified protein.
(2.34 MB TIF)

Figure S8 Peptide mass fingerprint spectrum of the protein in gel spot 13: Fumarate hydratase. Asterisks indicate fragment masses assigned to the identified protein.
(2.36 MB TIF)

Figure S9 Peptide mass fingerprint spectrum of the protein in gel spot 16: PSME1. Asterisks indicate fragment masses assigned to the identified protein.
(2.35 MB TIF)

Figure S10 Peptide mass fingerprint spectrum of the protein in gel spot 19: Aldose reductase. Asterisks indicate fragment masses assigned to the identified protein.
(2.39 MB TIF)

Figure S11 Peptide mass fingerprint spectrum of the protein in gel spot 21: HSP70 protein 9B. Asterisks indicate fragment masses assigned to the identified protein.
(2.28 MB TIF)

Figure S12 Peptide mass fingerprint spectrum of the protein in gel spot 23: Aconitase 2. Asterisks indicate fragment masses assigned to the identified protein.
(2.29 MB TIF)

Figure S13 Peptide mass fingerprint spectrum of the protein in gel spot 24: Aconitase 2. Asterisks indicate fragment masses assigned to the identified protein.
(2.29 MB TIF)

Figure S14 Peptide mass fingerprint spectrum of the protein in gel spot 27: hnRNP1. Asterisks indicate fragment masses assigned to the identified protein.
(2.27 MB TIF)

Figure S15 Peptide mass fingerprint spectrum of the protein in gel spot 29: VCP. Asterisks indicate fragment masses assigned to the identified protein.
(2.28 MB TIF)

Figure S16 Peptide mass fingerprint spectrum of the protein in gel spot 30: LDH H. Asterisks indicate fragment masses assigned to the identified protein.
(2.49 MB TIF)

Figure S17 Peptide mass fingerprint spectrum of the protein in gel spot 31: LAP3. Asterisks indicate fragment masses assigned to the identified protein.
(2.28 MB TIF)
