# Supplementary material for: Proteome Serological Determination of Tumor-Associated Antigens in Melanoma
Source: PLoS One. 2009 Apr 17;4(4):e5199. doi: 10.1371/journal.pone.0005199 (PMC2667248; doi:10.1371/journal.pone.0005199)
Supplement: Table S1 — Patient data for the sera used in the study. Melanoma patients and sera used for proteome-serological analysis of the tumor-associated antigenicity in melanoma. (0.22 MB DOC) [file pone.0005199.s001.doc]

| **#** | **Age** | **Sex** | **HLA-I** | **Diagnosis** | | **Stage** | **mm tumor thickness** |
| --- | --- | --- | --- | --- | --- | --- | --- |
| 1 | 84 | m | n.d.a. | SSM | 4 | | 4.5 |
| 2 | 70 | m | A1 | SSM | 4 | | 1.76 |
| 3 | 63 | m | A3 | MM | 4 | | - |
| 4 | n.d.a. | w | n.d.a. | MM | 4 | | - |
| 5 | 56 | m | n.d.a. | NM | 4 | | 2.16 |
| 6 | 61 | w | A2 A32 | SSM | 3-4 | | 1.25 |
| 7 | n.d.a. | m | n.d.a. | MM | 4 | | - |
| 8 | 76 | m | A1 A24 | NM | 4 | | 4.2 |
| 9 | 63 | m | A2 | SSM | 3 | | 0.85 |
| 10 | 72 | m | A3 | MM | 4 | | - |
| 11 | 65 | m | A26 | SSM | 3 | | 0.73 |
| 12 | 61 | w | A2 | MM | 4 | | - |
| 13 | 72 | m | n.d.a. | SSM | 2 | | 0.22 |
| 14 | 30 | m | A24 | MM | 4 | | - |
| 15 | 77 | w | A24 | MM | 4 | | - |
| 16 | 66 | w | n.d.a. | SSM | 3 | | 1.15 |
| 17 | n.d.a. | m | n.d.a. | MM | 4 | | - |
| 18 | n.d.a. | w | n.d.a. | MM | 4 | | - |
| 19 | n.d.a. | w | n.d.a. | MM | 4 | | - |
| 20 | 82 | w | A1 | LMM | 3 | | 0.86 |
| 21 | 81 | m | A2 | SSM | 3 | | 3.96 |
| 22 | 50 | m | n.d.a. | SSM | n.d.a. | | - |
| 23 | 55 | w | A2 | MM | 4 | | - |
| 24 | 66 | w | A1 | NM | 4 | | 7.8 |
| 25 | 66 | w | A3 | SSM | 3 | | 0.76 |
| 26 | 69 | m | A1 A25 | MM | 4 | | - |
| 27 | 39 | w | A1 | MM | 4 | | - |
| 28 | 34 | m | A24 | SSM | 4 | | 5.8 |
| 29 | 64 | m | A1 A11 | MM | 4 | | - |
| 30 | 24 | w | n.d.a. | MM | 4 | | 1.6 |
| 31 | 59 | w | A2 | SSM | 3 | | 0.5 |
| 32 | 65 | m | A2 | NM | 4 | | 1.29 |
| 33 | 63 | w | A1 A24 | SSM | 3 | | 1.54 |
| 34 | 80 | w | n.d.a. | MM | 3 | | - |
| 35 | 61 | w | A1 A2 B8 | SSM | 3 | | 0.91 |
| 36 | 74 | w | A2 | ALM | 4 | | 4.05 |
| 37 | 58 | m | n.d.a. | SSM | 4 | | 1.19 |
| 38 | 70 | w | n.d.a. | LMM | n.d.a. | | - |
| 39 | 72 | m | n.d.a. | MM | 4 | | - |
| 40 | 74 | w | A1 | MM | 4 | | - |
| 41 | 76 | w | A2 | SSM | 3 | | 0.67 |
| 42 | 57 | m | A1 A2 | SSM | 3 | | 0.92 |
| 43 | 61 | w | A2 | MM | 3 | | 0.99 |
| 44 | 70 | w | A3 | SSM | 3 | | 0.64 |
| 45 | 68 | m | n.d.a. | SSM | 3 | | 0.75 |
| 46 | 62 | m | A3 | MM | 4 | | 1.56 |
| 47 | 55 | w | A25 | SSM | n.d.a. | | - |
| 48 | 60 | m | A1 | SSM | 4 | | 1.26 |
| 49 | 67 | m | A2 | SSM | 3 | | 0.62 |
| 50 | 60 | w | A11 | MM | 3 | | 0.84 |

| **#** | **Age** | **Sex** | **HLA-I** | **Diagnosis** | | **Stage** | **mm tumor thickness** |
| --- | --- | --- | --- | --- | --- | --- | --- |
| 51 | 45 | w | A2 | SSM | 4 | | 3.11 |
| 52 | 45 | w | n.d.a. | *In situ* NM | *In situ* | | - |
| 53 | 41 | m | A1 A23 | MM | 4 | | 8 |
| 54 | 72 | m | A2 | SSM | 3 | | 0.47 |
| 55 | 81 | w | n.d.a. | SSM | 4 | | 1.85 |
| 56 | 91 | w | A29 | LMM | n.d.a. | | - |
| 57 | 53 | w | A1 | MM | 3 | | - |
| 58 | 28 | w | A2 | NM | 4 | | 2.2 |
| 59 | 76 | w | A2 | NM | 4 | | 2 |
| 60 | 68 | m | A25 | SSM | 2 | | 0.5 |
| 61 | 72 | w | A3 | NM | 4 | | 3 |
| 62 | 84 | w | A3 | MM | 4 | | - |
| 63 | 70 | m | A1 A2 B7 | MM | 4 | | - |
| 64 | 54 | w | A2 | MM | 3 | | 1.08 |
| 65 | 63 | w | A11 | SSM | 3 | | 0.59 |
| 66 | 65 | m | A11 | SSM | 3 | | 0.45 |
| 67 | 52 | m | n.d.a. | MM | 4 | | - |
| 68 | 77 | m | A2 | SSM | 4 | | 7.5 |
| 69 | 47 | w | A1 | NM | 3 | | 1 |
| 70 | 55 | w | A2 | MM | 3 | | 0.68 |
| 71 | 59 | m | n.d.a. | MM | 4 | | - |
| 72 | 42 | w | A1 | NM | 4 | | 3.15 |
| 73 | 39 | m | A2 A3 | NM | 4 | | 3 |
| 74 | 56 | w | A2 | NM | 4 | | 2.7 |
| 75 | 42 | m | A10 | MM | 4 | | - |
| 76 | n.d.a. | w | A2 | NM | 4 | | 0.8 |
| 77 | 62 | w | A2 | ALM | 2 | | 0.56 |
| 78 | n.d.a. | m | n.d.a. | MM | 4 | | - |
| 79 | 43 | w | A2 | SSM | 3 | | 0.75 |
| 80 | n.d.a. | m | n.d.a. | MM | 3 | | - |
| 81 | 40 | w | A2 | SSM | 3 | | 0.64 |
| 82 | 53 | w | A11 | SSM | n.d.a. | | - |
| 83 | 43 | w | A24 | MM | 4 | | - |
| 84 | 62 | w | A23 | MM | 4 | | - |
| 85 | 69 | m | A2 A3 | MM | 4 | | - |
| 86 | 67 | m | n.d.a. | SSM | 3 | | 0.6 |
| 87 | 64 | m | A3 A24 | MM | 3 | | - |
| 88 | 62 | m | A3 | MM | 4 | | - |
| 89 | 54 | w | A2 | ALM | 3 | | 1.08 |
| 90 | 67 | w | n.d.a. | SSM | 4 | | 2.1 |
| 91 | 90 | w | n.d.a. | NM | 4 | | 6 |
| 92 | n.d.a. | m | n.d.a. | MM | 4 | | - |
| 93 | n.d.a. | m | n.d.a. | MM | n.d.a. | | - |
| 94 | n.d.a. | w | n.d.a. | MM | 3 | | - |
| 95 | n.d.a. | m | n.d.a. | MM | n.d.a. | | - |

SSM, superfacially spreading melanoma; NM, nodular melanoma; ALM, acral lentiginous melanoma; LMM, lentigo maligna melanoma; MM, metastatic melanoma; n.d.a., no data available.
