# Supplementary figures and images for: Proteome Serological Determination of Tumor-Associated Antigens in Melanoma
Source: PLoS One. 2009 Apr 17;4(4):e5199. doi: 10.1371/journal.pone.0005199 (PMC2667248; doi:10.1371/journal.pone.0005199)

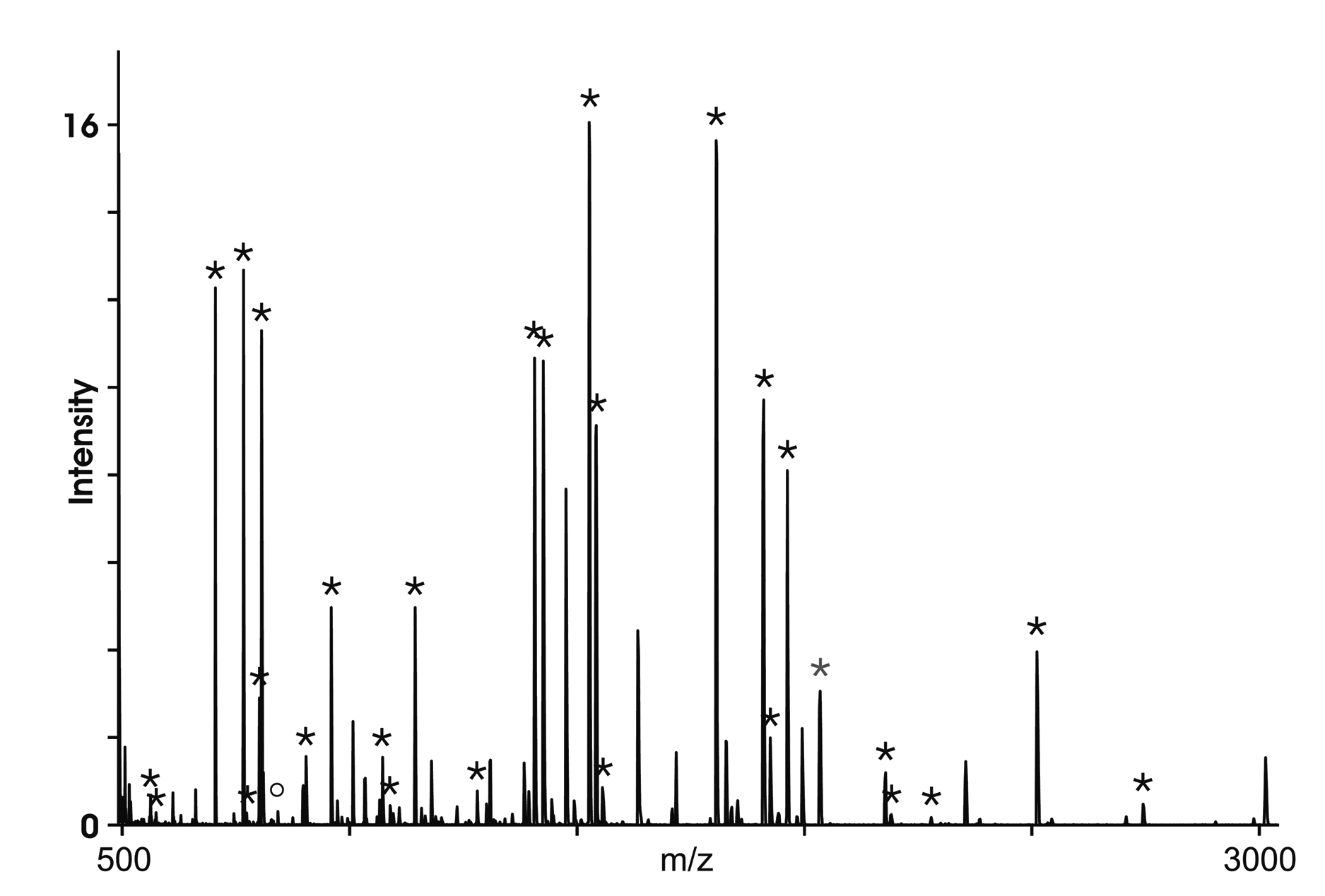

Supplement: Figure S1 — Peptide mass fingerprint spectrum of the protein in gel spot 2: Enolase 1. Asterisks indicate fragment masses assigned to the identified protein. (2.40 MB TIF) [file pone.0005199.s002.tif]

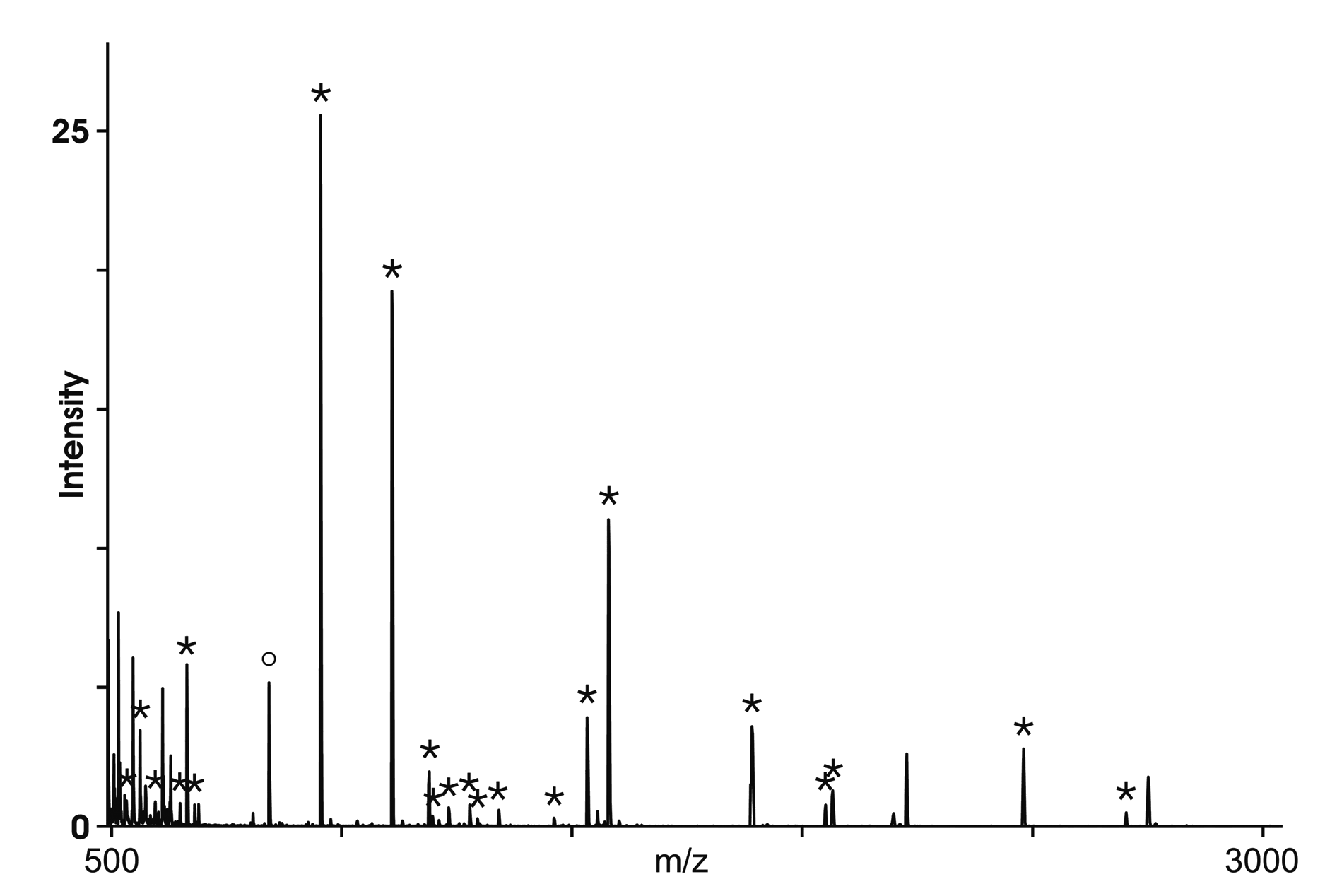

Supplement: Figure S2 — Peptide mass fingerprint spectrum of the protein in gel spot 3: Calumenin. Asterisks indicate fragment masses assigned to the identified protein. (2.40 MB TIF) [file pone.0005199.s003.tif]

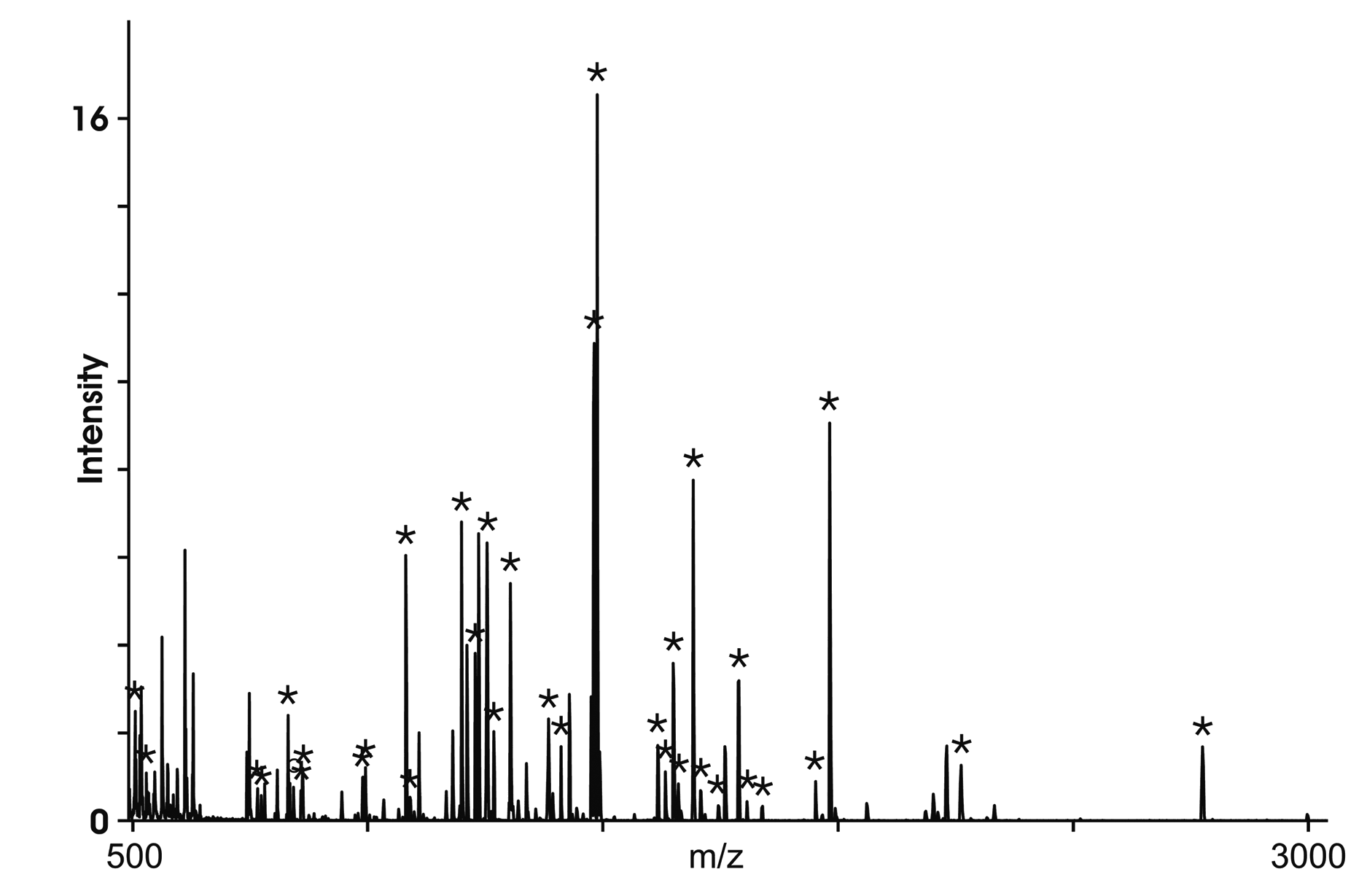

Supplement: Figure S3 — Peptide mass fingerprint spectrum of the protein in gel spot 5: HSP70 protein B. Asterisks indicate fragment masses assigned to the identified protein. (2.32 MB TIF) [file pone.0005199.s004.tif]

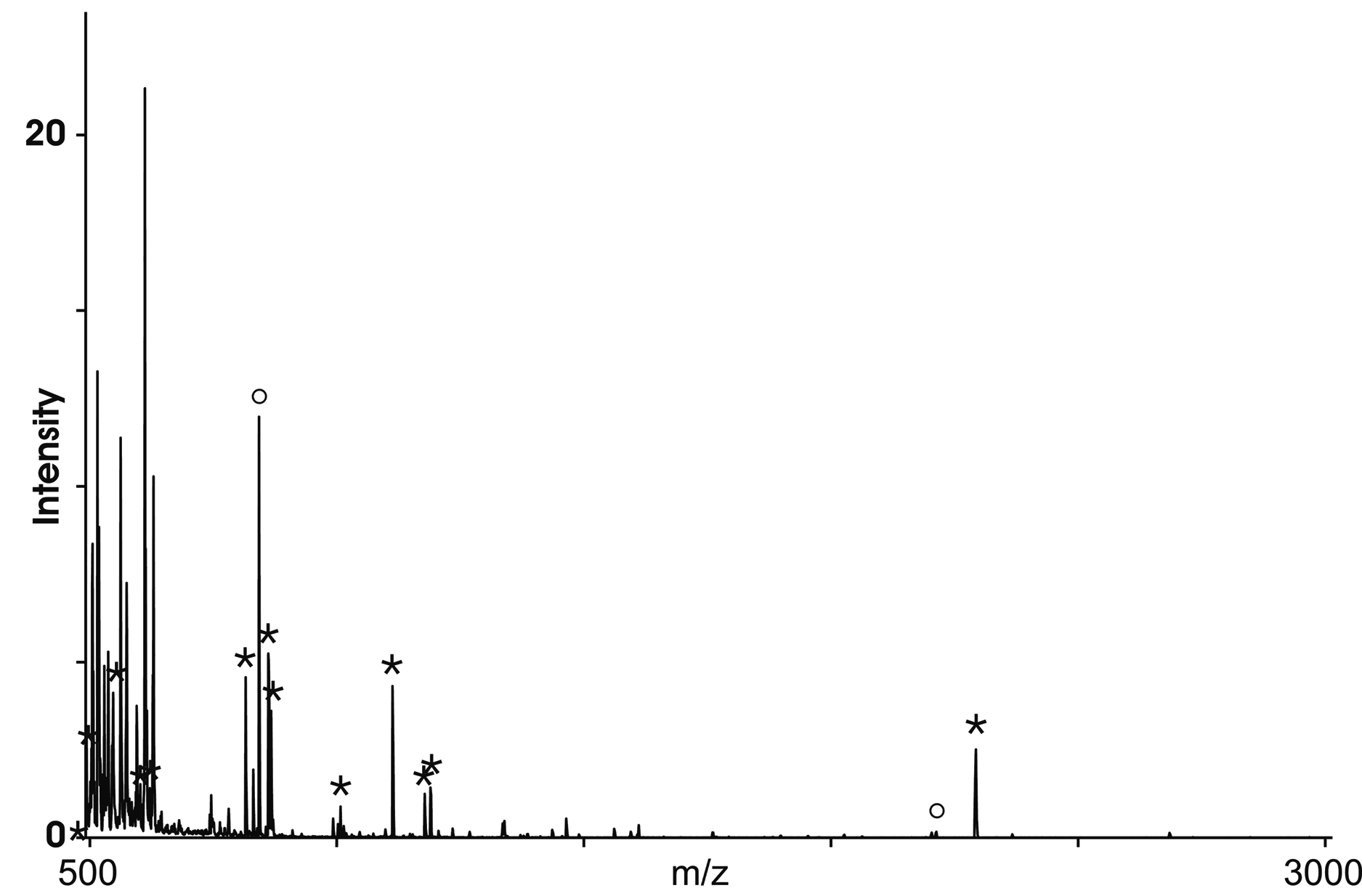

Supplement: Figure S4 — Peptide mass fingerprint spectrum of the protein in gel spot 8: 2,4-dienoyl-CoA reductase. Asterisks indicate fragment masses assigned to the identified protein. (2.33 MB TIF) [file pone.0005199.s005.tif]

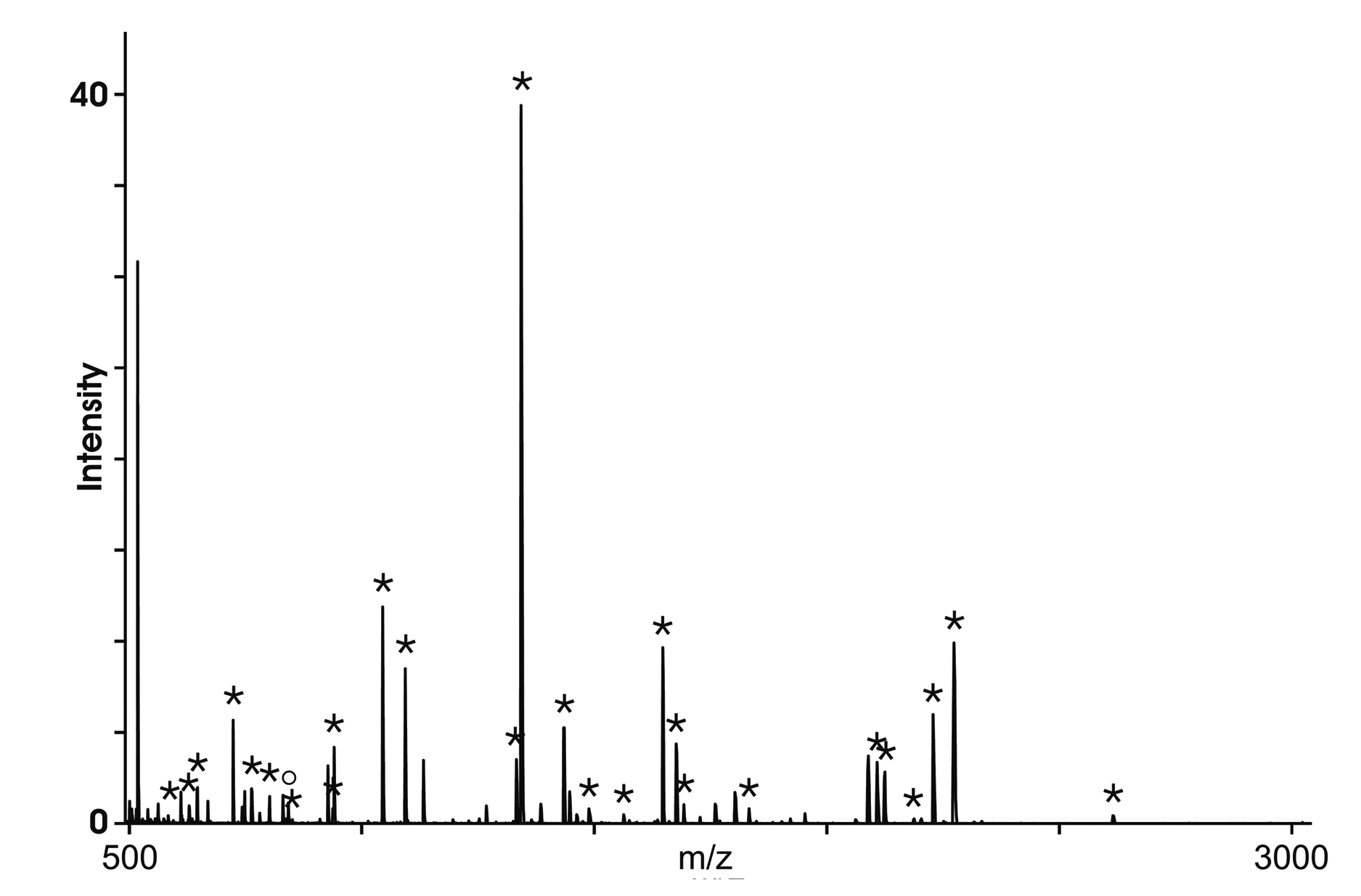

Supplement: Figure S5 — Peptide mass fingerprint spectrum of the protein in gel spot 10: Aldolase A. Asterisks indicate fragment masses assigned to the identified protein. (2.34 MB TIF) [file pone.0005199.s006.tif]

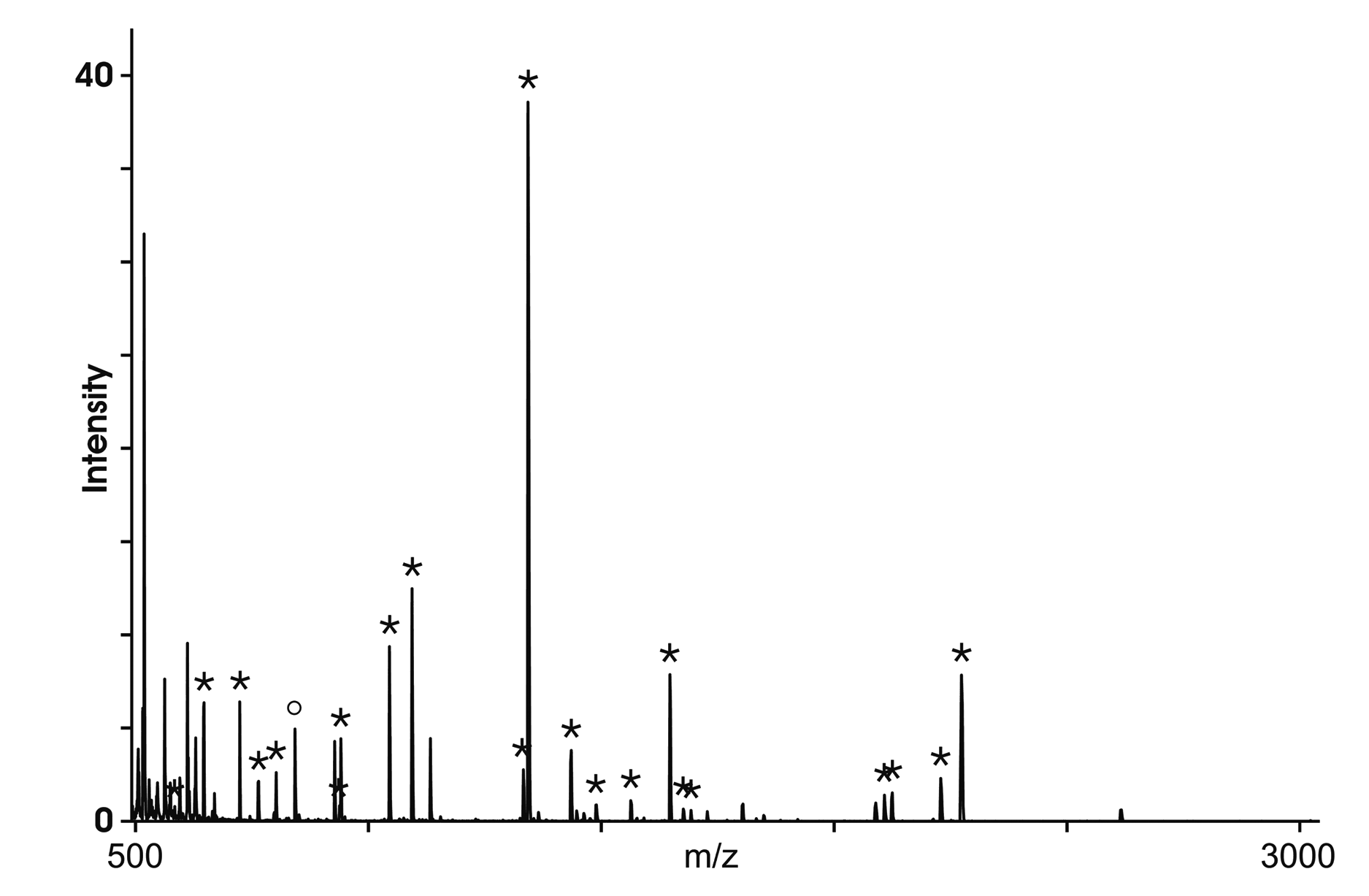

Supplement: Figure S6 — Peptide mass fingerprint spectrum of the protein in gel spot 11: Aldolase A. Asterisks indicate fragment masses assigned to the identified protein. (2.32 MB TIF) [file pone.0005199.s007.tif]

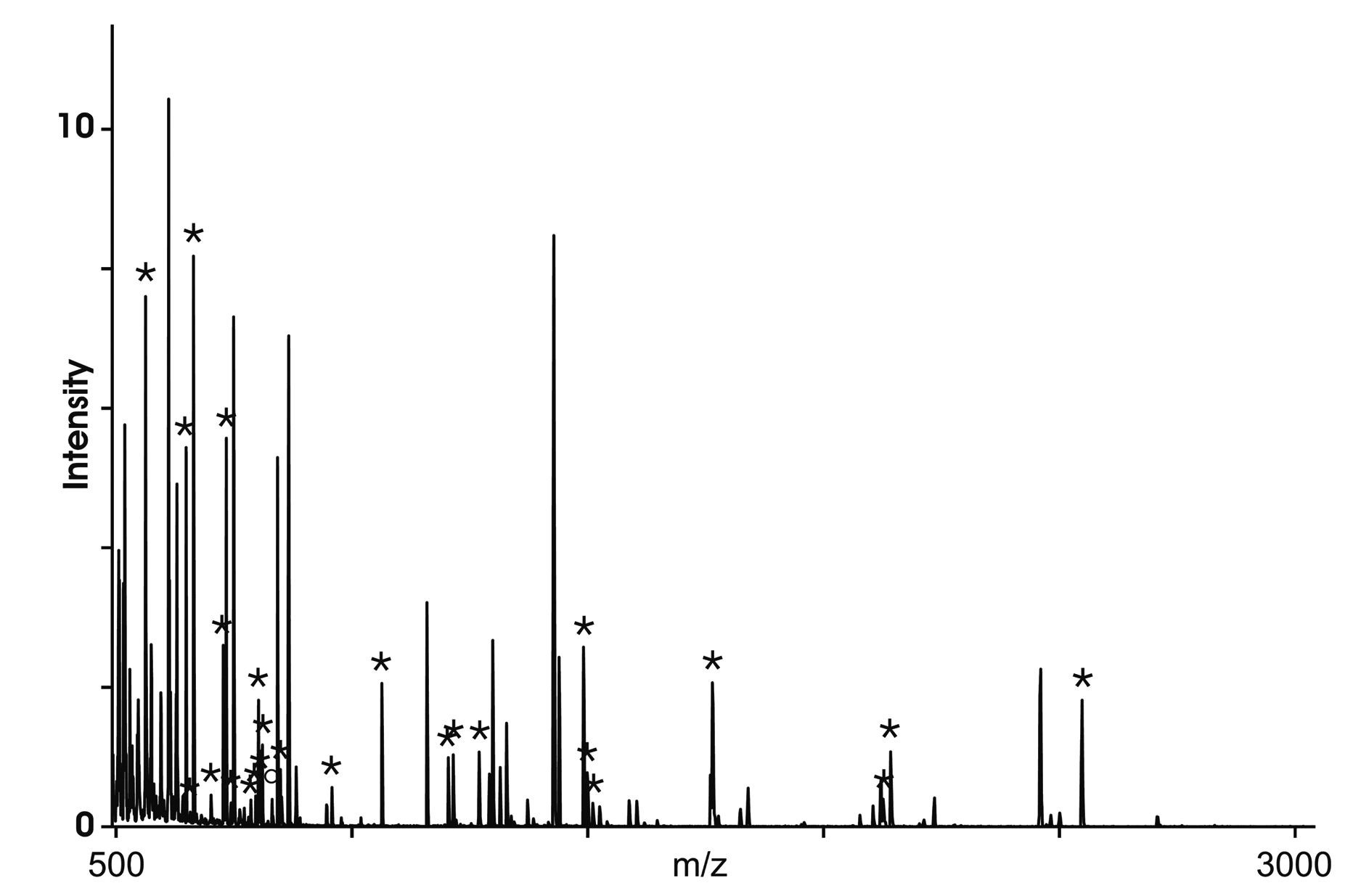

Supplement: Figure S7 — Peptide mass fingerprint spectrum of the protein in gel spot 12: Fumarate hydratase. Asterisks indicate fragment masses assigned to the identified protein. (2.34 MB TIF) [file pone.0005199.s008.tif]

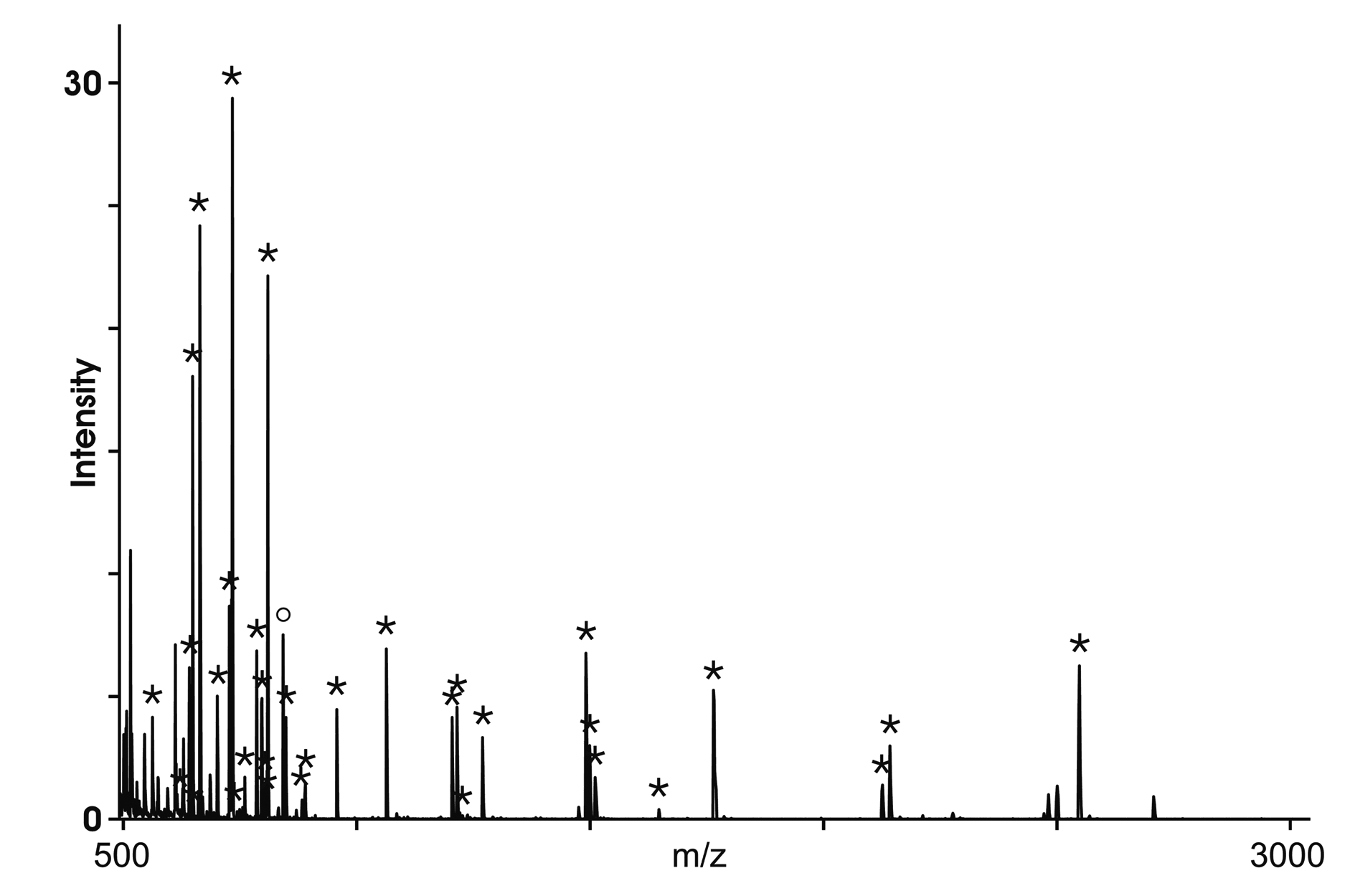

Supplement: Figure S8 — Peptide mass fingerprint spectrum of the protein in gel spot 13: Fumarate hydratase. Asterisks indicate fragment masses assigned to the identified protein. (2.36 MB TIF) [file pone.0005199.s009.tif]

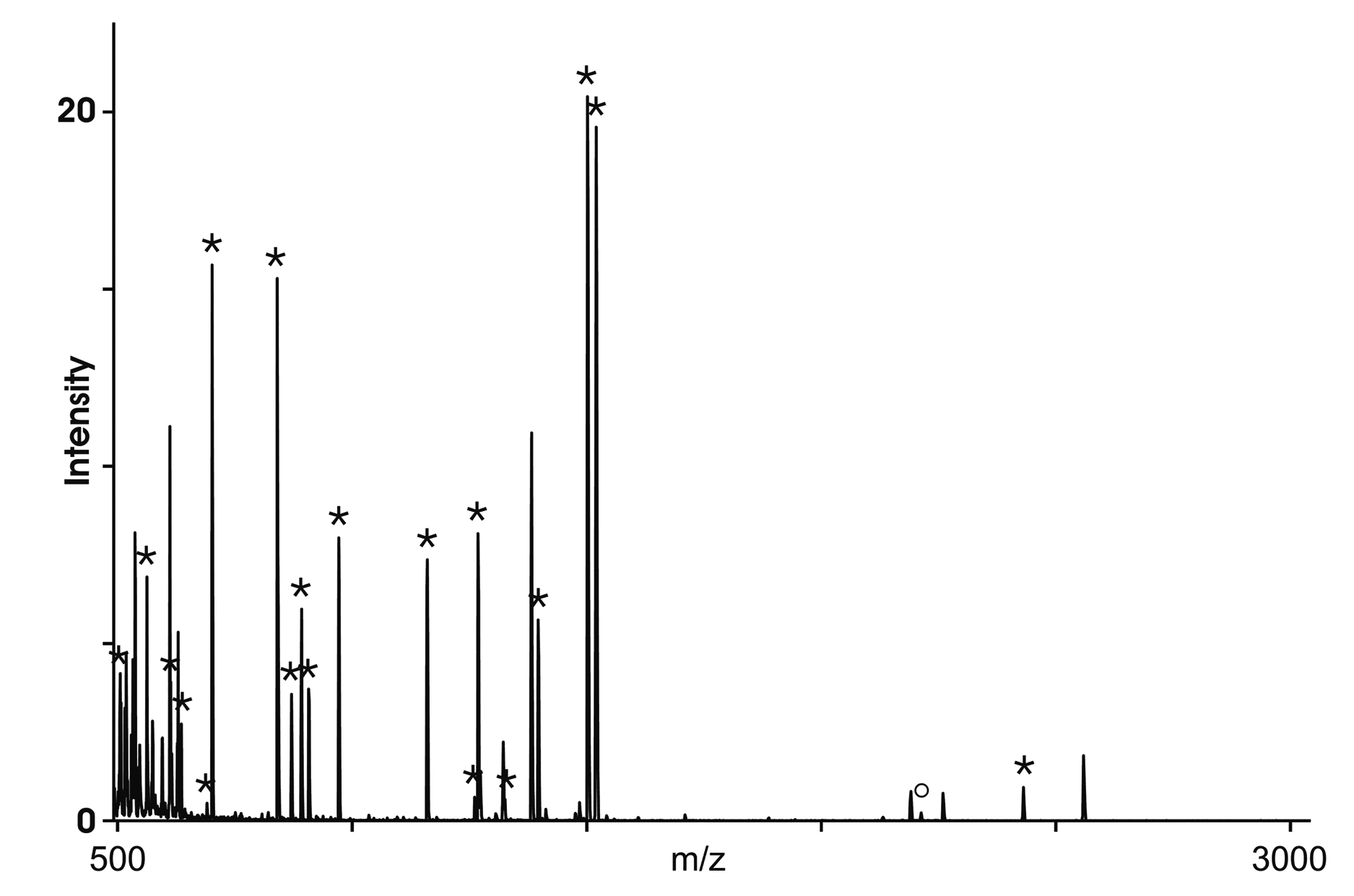

Supplement: Figure S9 — Peptide mass fingerprint spectrum of the protein in gel spot 16: PSME1. Asterisks indicate fragment masses assigned to the identified protein. (2.35 MB TIF) [file pone.0005199.s010.tif]

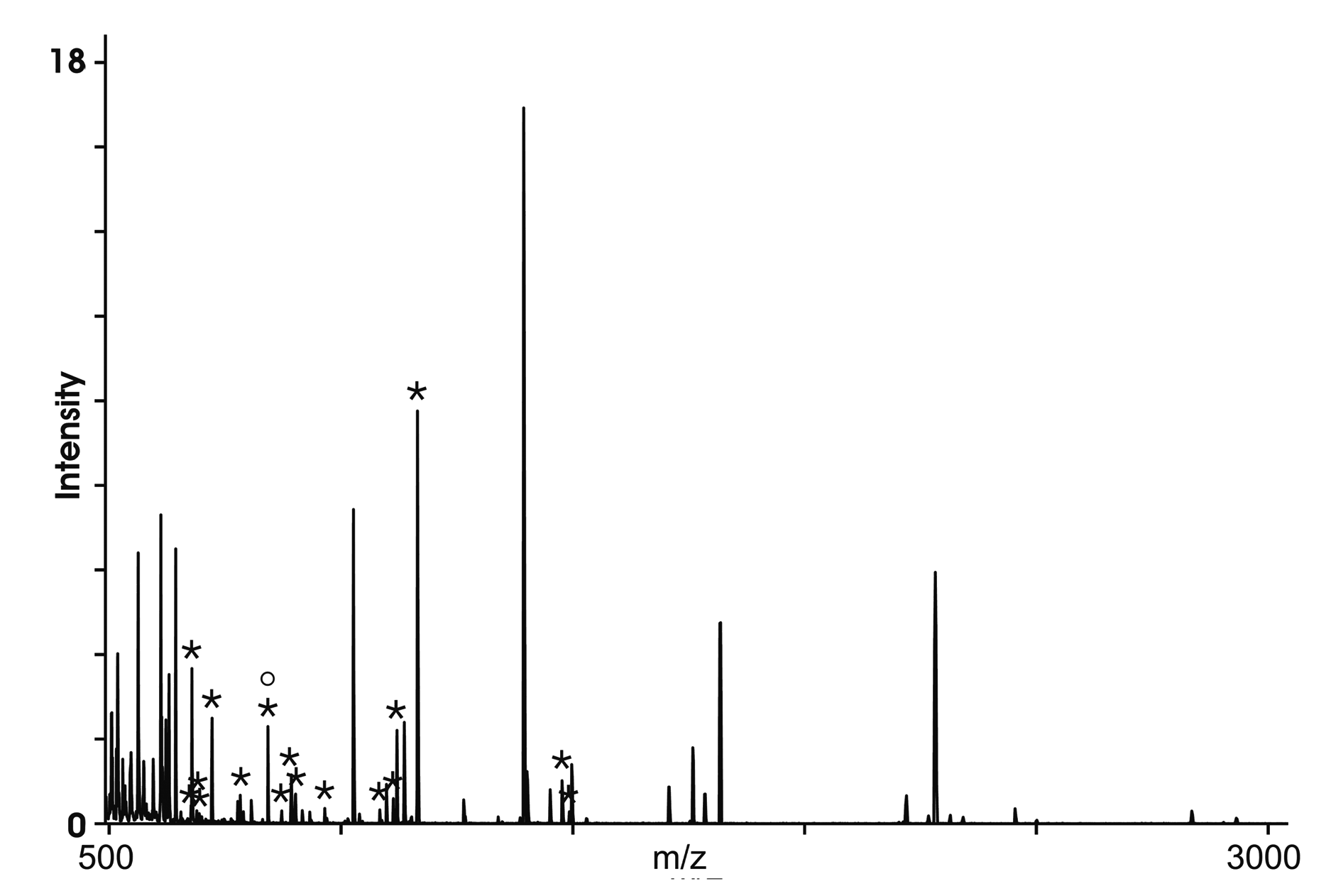

Supplement: Figure S10 — Peptide mass fingerprint spectrum of the protein in gel spot 19: Aldose reductase. Asterisks indicate fragment masses assigned to the identified protein. (2.39 MB TIF) [file pone.0005199.s011.tif]

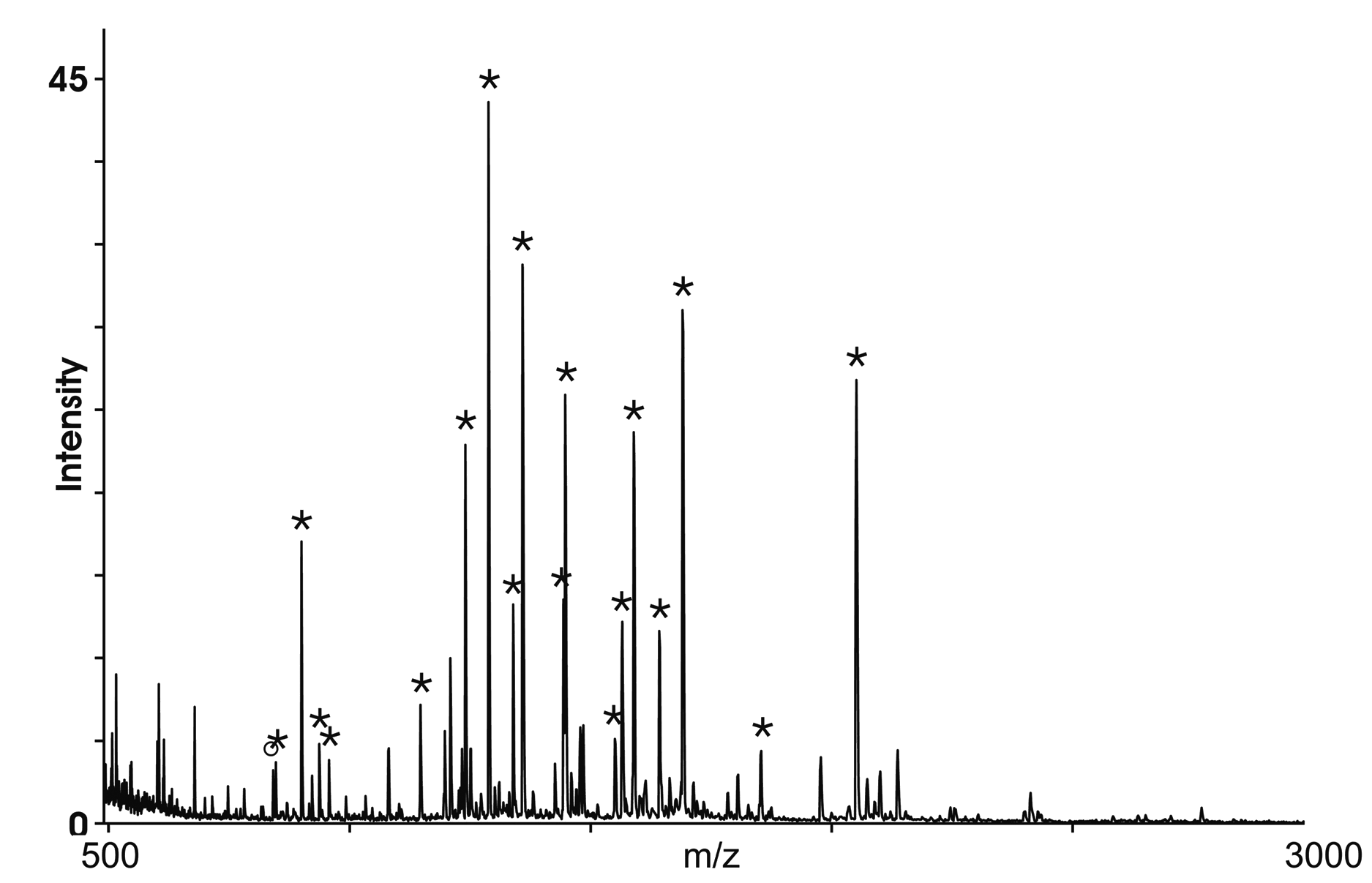

Supplement: Figure S11 — Peptide mass fingerprint spectrum of the protein in gel spot 21: HSP70 protein 9B. Asterisks indicate fragment masses assigned to the identified protein. (2.28 MB TIF) [file pone.0005199.s012.tif]

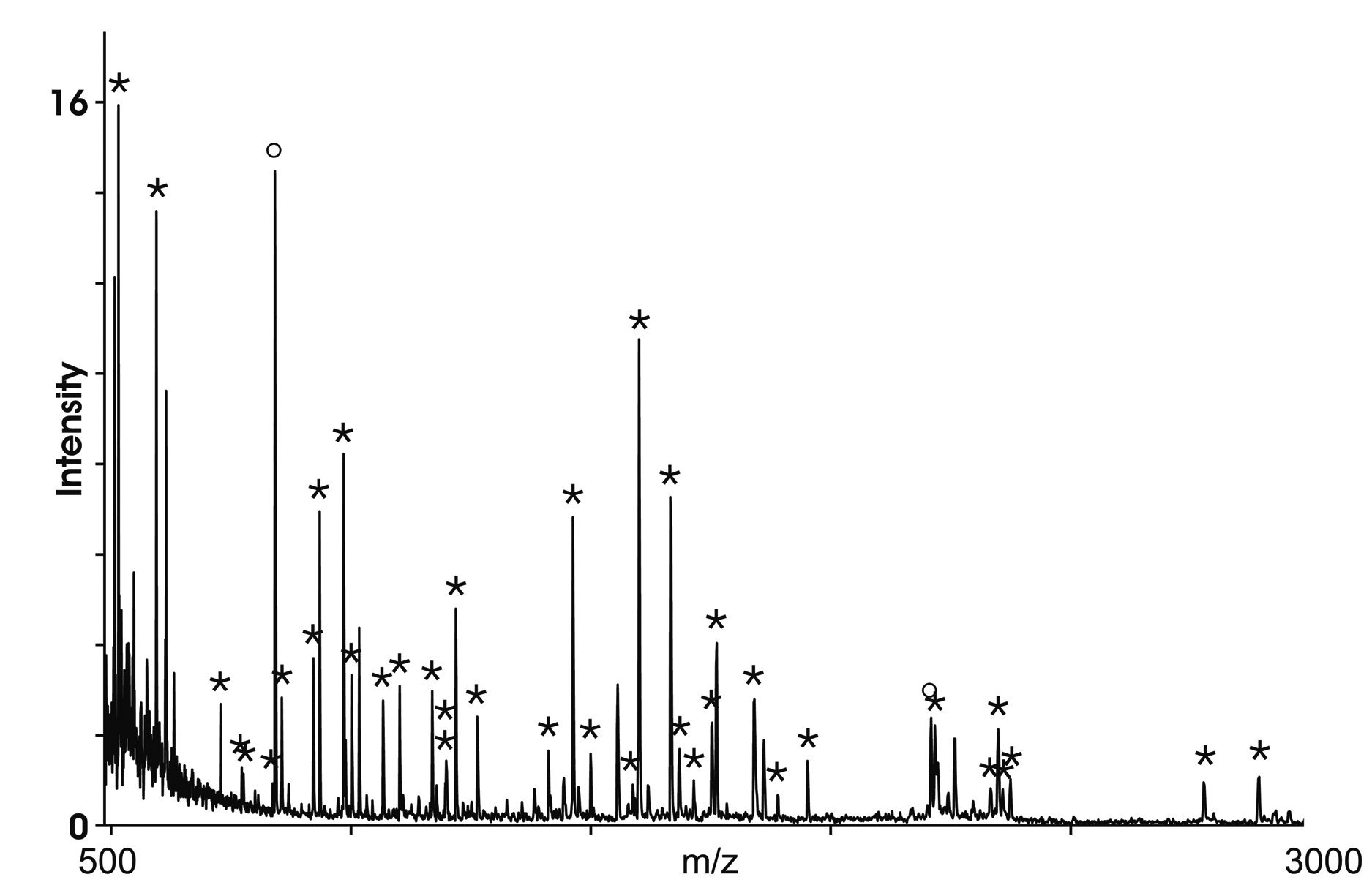

Supplement: Figure S12 — Peptide mass fingerprint spectrum of the protein in gel spot 23: Aconitase 2. Asterisks indicate fragment masses assigned to the identified protein. (2.29 MB TIF) [file pone.0005199.s013.tif]

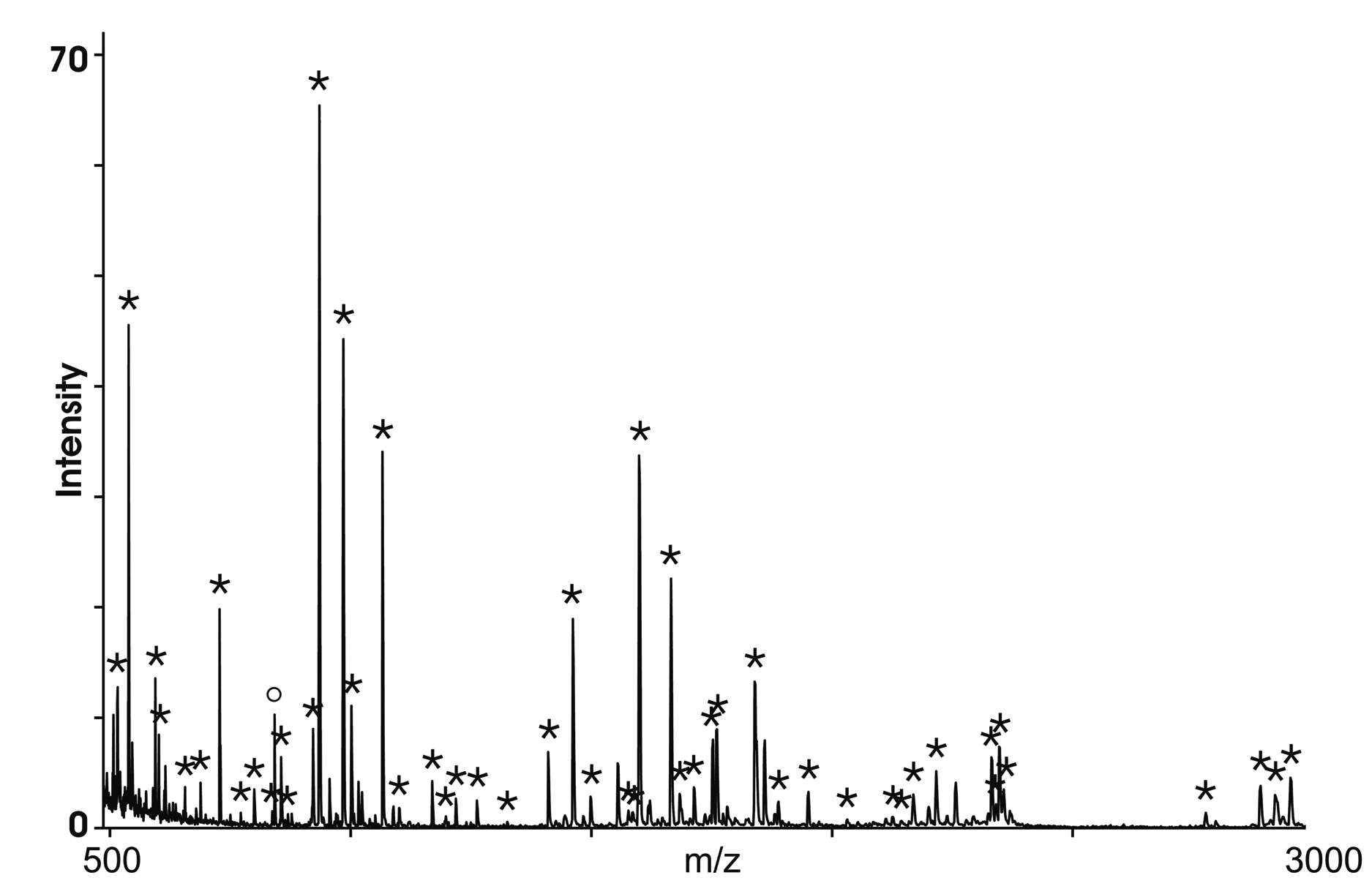

Supplement: Figure S13 — Peptide mass fingerprint spectrum of the protein in gel spot 24: Aconitase 2. Asterisks indicate fragment masses assigned to the identified protein. (2.29 MB TIF) [file pone.0005199.s014.tif]

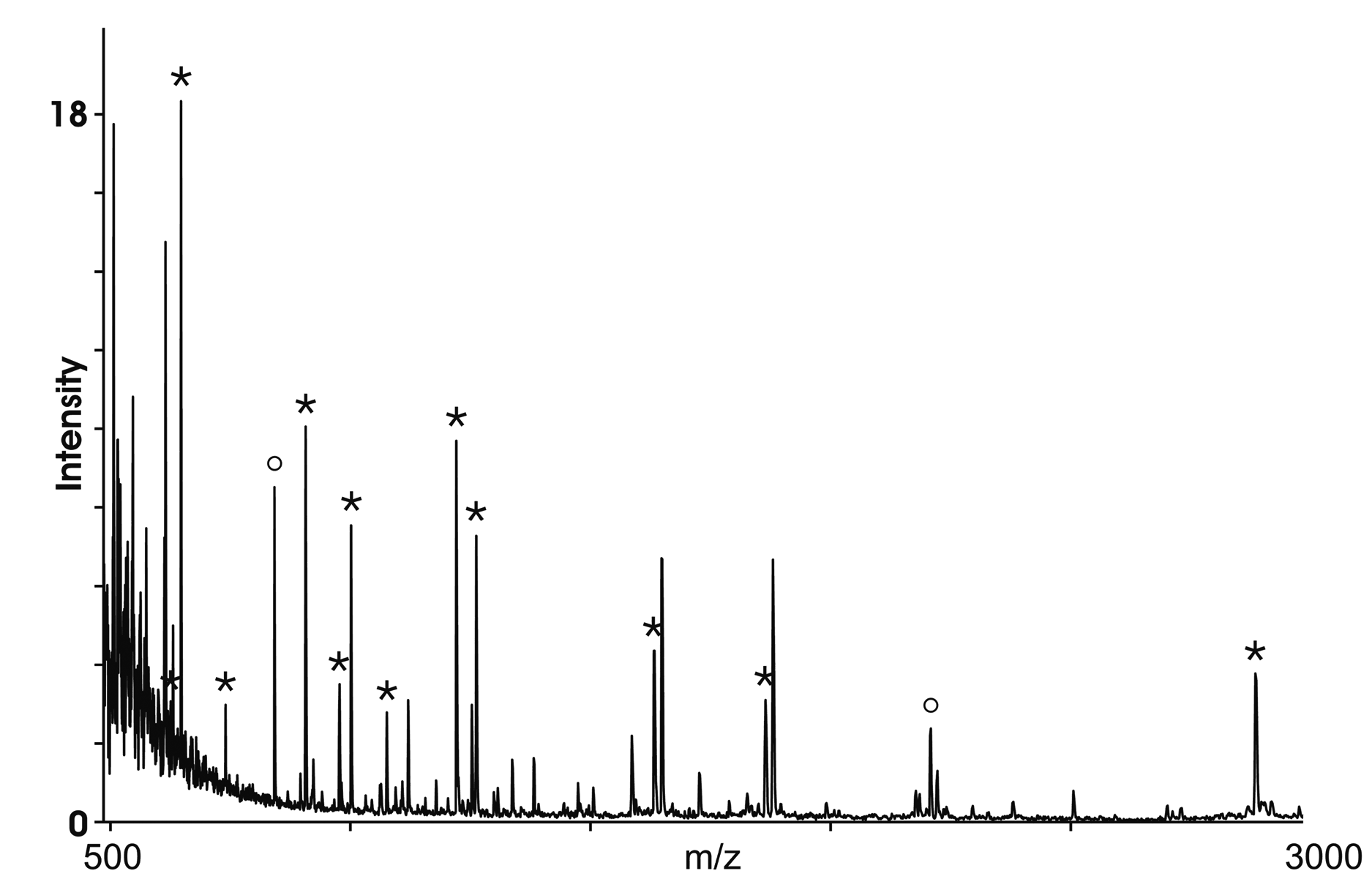

Supplement: Figure S14 — Peptide mass fingerprint spectrum of the protein in gel spot 27: hnRNP1. Asterisks indicate fragment masses assigned to the identified protein. (2.27 MB TIF) [file pone.0005199.s015.tif]

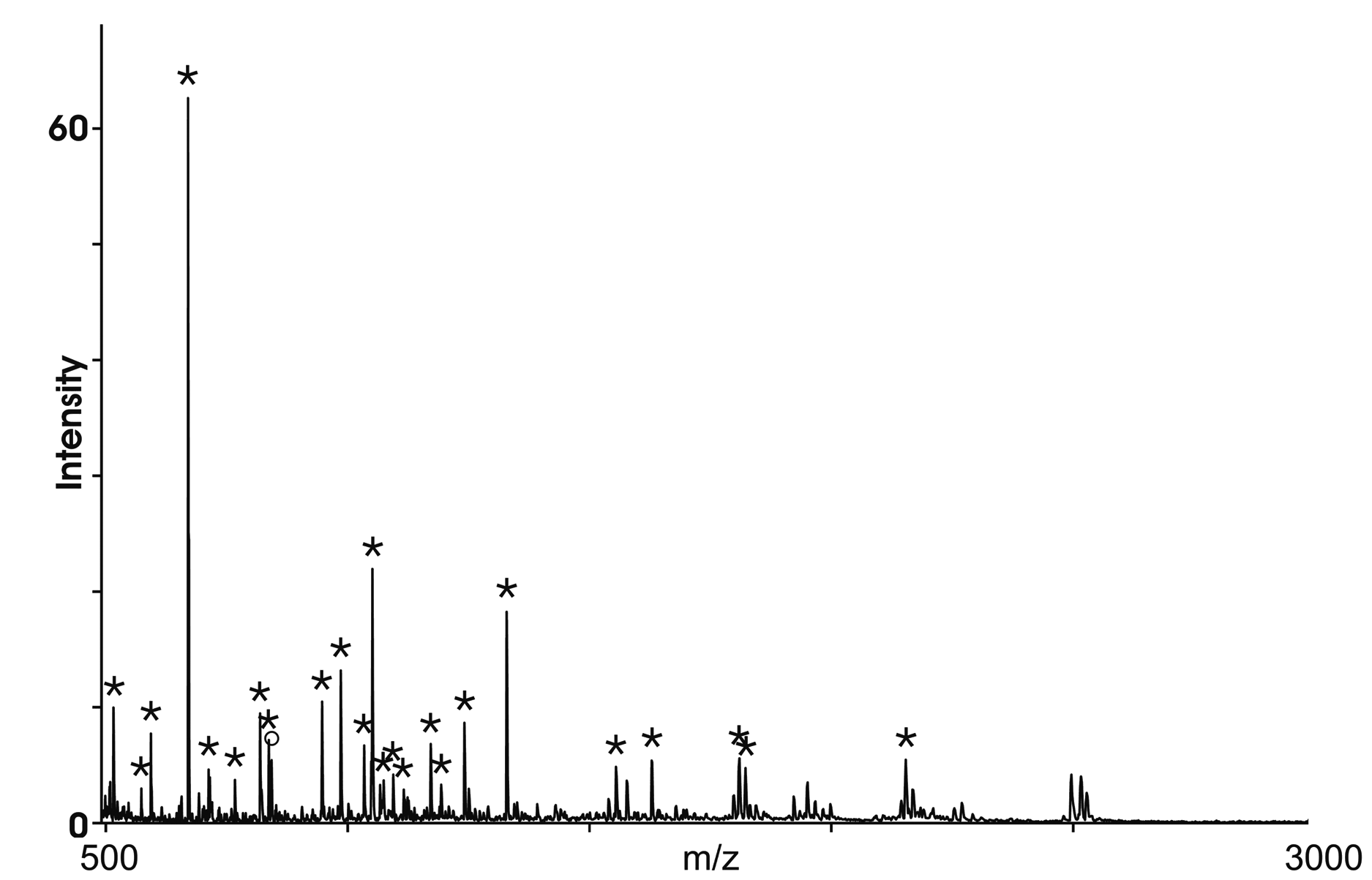

Supplement: Figure S15 — Peptide mass fingerprint spectrum of the protein in gel spot 29: VCP. Asterisks indicate fragment masses assigned to the identified protein. (2.28 MB TIF) [file pone.0005199.s016.tif]

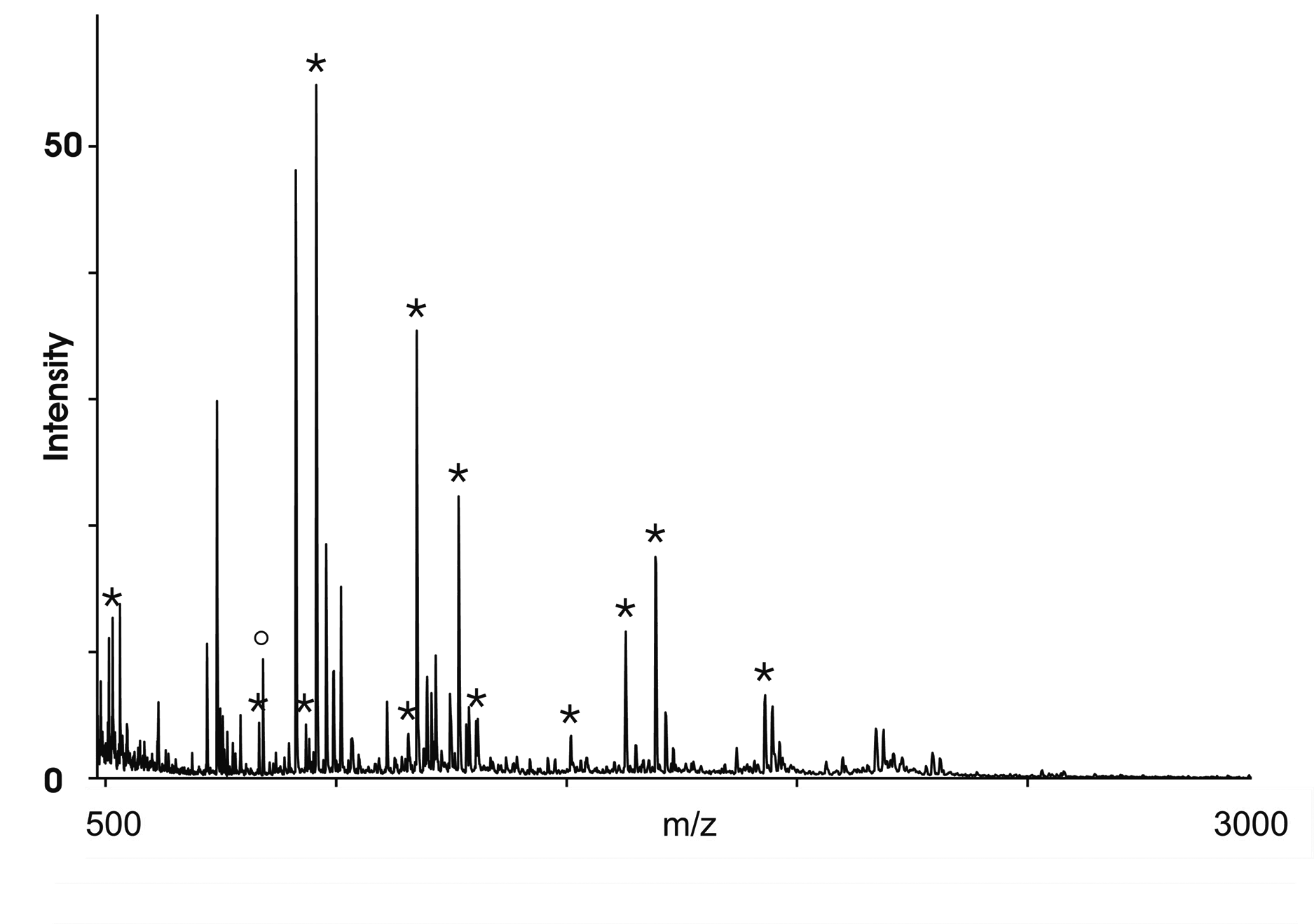

Supplement: Figure S16 — Peptide mass fingerprint spectrum of the protein in gel spot 30: LDH H. Asterisks indicate fragment masses assigned to the identified protein. (2.49 MB TIF) [file pone.0005199.s017.tif]

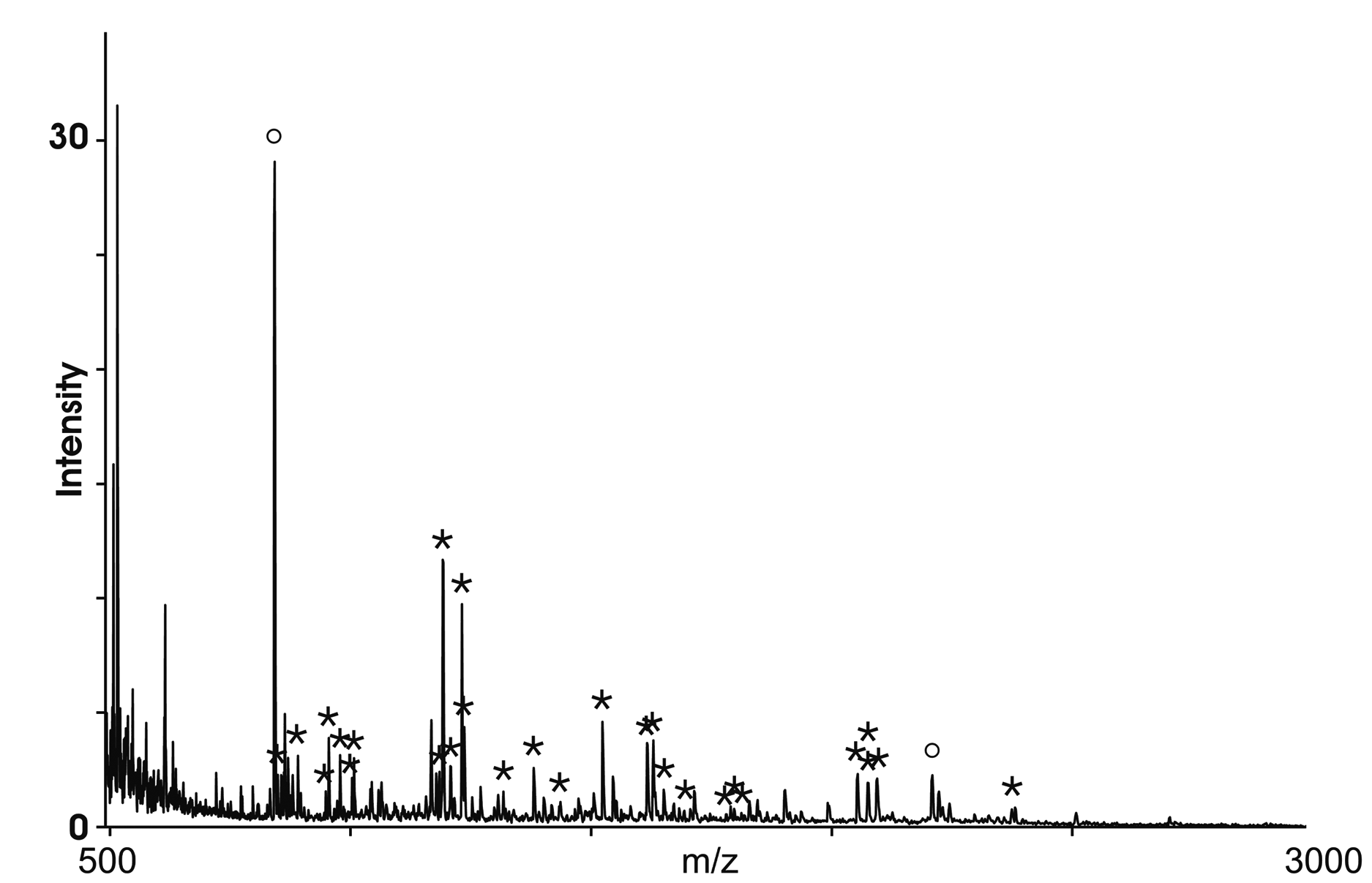

Supplement: Figure S17 — Peptide mass fingerprint spectrum of the protein in gel spot 31: LAP3. Asterisks indicate fragment masses assigned to the identified protein. (2.28 MB TIF) [file pone.0005199.s018.tif]
